# High-dimensional immunotyping of tumors grown in obese and non-obese mice

**DOI:** 10.1242/dmm.048977

**Published:** 2021-03-30

**Authors:** Cara E. Wogsland, Hilde E. Lien, Line Pedersen, Pahul Hanjra, Sturla M. Grondal, Rolf A. Brekken, James B. Lorens, Nils Halberg

**Affiliations:** 1 Department of Biomedicine, University of Bergen, N-5020 Bergen, Norway; 2 Division of Surgical Oncology, Department of Surgery, and Hamon Center for Therapeutic Oncology Research, University of Texas Southwestern Medical Center, Dallas, TX 75390, USA; 3 Department of Pharmacology, University of Texas Southwestern Medical Center, Dallas, TX 75390, USA

**Keywords:** Tumor immunology, Suspension mass cytometry, Batch correction, Obesity, Breast cancer, Pancreatic cancer

## Abstract

Obesity is a disease characterized by chronic low-grade systemic inflammation and has been causally linked to the development of 13 cancer types. Several studies have been undertaken to determine whether tumors evolving in obese environments adapt differential interactions with immune cells and whether this can be connected to disease outcome. Most of these studies have been limited to single-cell lines and tumor models and analysis of limited immune cell populations. Given the multicellular complexity of the immune system and its dysregulation in obesity, we applied high-dimensional suspension mass cytometry to investigate how obesity affects tumor immunity. We used a 36-marker immune-focused mass cytometry panel to interrogate the immune landscape of orthotopic syngeneic mouse models of pancreatic and breast cancer. Unanchored batch correction was implemented to enable simultaneous analysis of tumor cohorts to uncover the immunotypes of each cancer model and reveal remarkably model-specific immune regulation. In the E0771 breast cancer model, we demonstrate an important link to obesity with an increase in two T-cell-suppressive cell types and a decrease in CD8 T cells.

## Abstract (precis)

**Summary:** We used a mass cytometry analysis pipeline to immunophenotype tumor immune infiltrate from two pancreatic and three breast cancer murine models, identifying a link to obesity in the E0771 breast cancer model.

## INTRODUCTION

Obesity is a risk factor for at least 13 types of cancer including breast and pancreatic cancer (Genkinger et al., 2011; Pierobon and Frankenfeld, 2013; Renehan et al., 2015; Picon-Ruiz et al., 2017). In addition to being associated with risk of cancer, obesity correlates with worse prognosis and higher mortality rates among breast and pancreatic cancer patients (Yuan et al., 2013; Choi et al., 2016; Chan and Norat, 2015; Calle et al., 2003; Chan et al., 2014). The mechanisms by which obesity contributes to cancer development and outcome are currently incompletely understood (Donohoe et al., 2017). Obesity leads to local and systemic inflammation and immune system dysregulation, characterized by increased levels of pro-inflammatory cytokines and pro-inflammatory and immunosuppressive immune cells (Apostolopoulos et al., 2016). Specific obesity-induced changes include increased abundance of immunosuppressive myeloid-derived suppressor cells (MDSCs), pro-inflammatory M1 and metabolically activated macrophages, and associated crown-like structures found in obese adipose tissue (Coats et al., 2017; Kratz et al., 2014; Pawelec et al., 2019; Hale et al., 2015; Weisberg et al., 2003; Tiwari et al., 2019; Xu et al., 2003). Breast and pancreatic tumors are likely to be impacted by the local and systemic effects of obesity because these tumors develop in close proximity to mammary and omental adipose tissue, respectively.

Immunocompetent models of obesity and cancer are necessary to study immune changes in cancer in the obese environment. The C57Bl/6 mouse strain has been shown to have an obese phenotype when fed a high-fat diet (HFD), including increased body mass, elevated blood sugar levels and insulin resistance (Tiwari et al., 2019; Coats et al., 2017). When paired with such diet-induced obesity (DIO), syngeneic tumor cell lines can be used to study cancer-associated immune system changes during obesity. Increased tumor incidence and accelerated tumor growth have been demonstrated in multiple obese murine models (Incio et al., 2016a; Khasawneh et al., 2009; Tiwari et al., 2019; Cranford et al., 2019; Wang et al., 2019; Chung et al., 2020; Qureshi et al., 2020; Incio et al., 2016b). However, the immune cell compartment of the tumor microenvironment and its possible impact on obesity-induced tumors has not been systematically characterized at the single-cell level.

Cancer progression is an evolutionary process in which the fitness of cancer cells is dependent on reciprocal interactions between tumor-intrinsic and -extrinsic factors, including immune cells. In particular, CD8 T cells are central to tumor immunity, and tumor-infiltrating CD8 T cells are associated with increased patient survival (Martínez-Lostao et al., 2015; Liu et al., 2012; Mahmoud et al., 2012). In the tumor microenvironment, MDSCs possess strong T-cell-suppressive capacity, inhibiting T-cell function and proliferation (Bronte et al., 2016; Parker et al., 2015). Tumor-associated macrophages with the often oversimplified M1/M2 characterization – along with T cells, natural killer (NK) cells, dendritic cells (DCs), B cells and eosinophils – have complex and often inconsistent functions in cancer (Gonzalez et al., 2018; Varricchi et al., 2017; Wylie et al., 2019; Noy and Pollard, 2014).

Because of this intricate immune cell composition, conventional methods fail to reach the number of parameters required to profile the tumor immune microenvironment. High-dimensional single-cell approaches, such as mass cytometry, enable the simultaneous characterization of these varied cell types with multi-dimensional resolution.

Suspension mass cytometry (CyTOF) analysis of dissociated tumors can detect the multiple immune cell subsets required for an in-depth tumor immunotyping, but these datasets tend to be large and time consuming to collect. It is common to have datasets in multiple batches that are prepared, stained and collected on different days, owing to the length of time needed to process and stain the samples and subsequently collect the data. Palladium-based metal barcoding of up to 20 samples has allowed for simultaneous collection of multiple samples without run/batch differences (Zunder et al., 2015). This is a great improvement; however, many studies are composed of more than 20 samples. Therefore, it is common to end up with data in multiple batches that need to be compared. Having a common, or anchor, sample used in every batch can assist in the removal of batch effects but is not always available.

Here, we have implemented a robust mass cytometry analysis pipeline to correct for batch effects between unanchored batches with multistep clustering to maximize phenotyping and minimize bias. We have immunophenotyped tumor immune infiltrate from two syngeneic pancreatic and three syngeneic breast cancer models and present the data as an immunotype atlas containing 21 immune cell metaclusters present across the five tumor models. Additionally, we report immunotyping of tumors grown in obese mice for all five models. Our findings demonstrate that the tumor immune infiltrate composition is highly model and cancer type specific. One model, E0771 tumors, had significant immune cell differences between lean and obese mice. This breast cancer model showed an increase in G-MDSCs and PD-L1^+^ (also known as CD274^+^) DCs and a decrease in CD8 T cells in tumors from obese mice, making it a clinically relevant model (Jin and Hu, 2020; Mahmoud et al., 2012; Mahmoud et al., 2011).

## RESULTS

### Tumor immune infiltrating cells identified for seven CyTOF batches from obese and non-obese mice

**Fig. 1.**
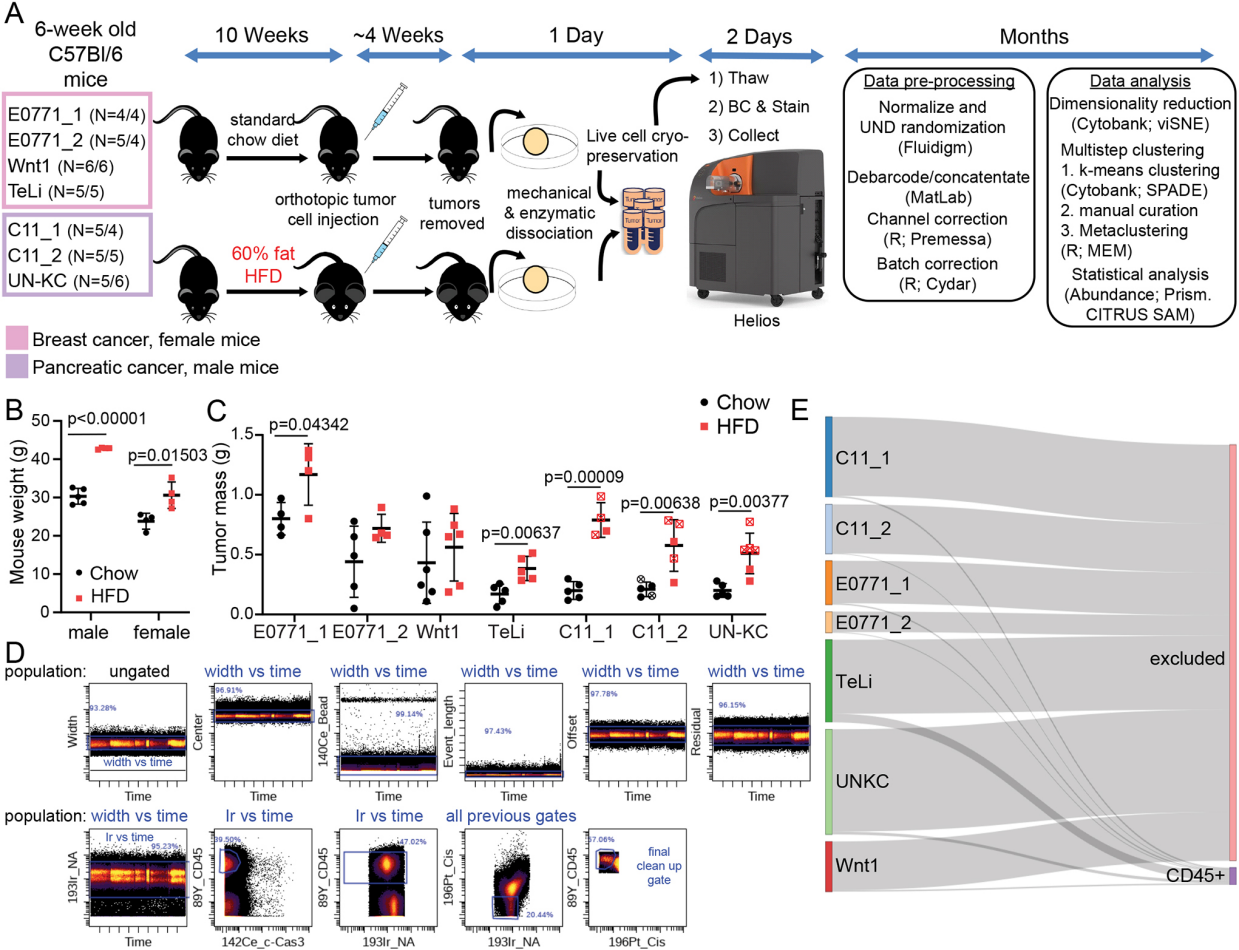
**Experimental design and analysis pipeline for mass cytometry data for immune infiltrate of seven CyTOF batches from five murine tumor models.** (A) Cartoon and timeline of experimental design, data collection, data preprocessing and analysis. For each batch, *n*=chow/HFD. E0771_1 (*n*=4/4), E0771_2 (*n*=5/4), Wnt1 (*n*=6/6), TeLi (*n*=5/5), C11_1 (*n*=5/4), C11_2 (*n*=5/5), UN-KC (*n*=5/6). UND, uniform negative distribution. For B and C, each scatter dot plot point is from a different animal (mean±s.d.). Unpaired Student's *t*-tests were not adjusted, with s.d. assumed. (B) Representative mouse weights for male (*n*=5/4) and female (*n*=5/4) C57Bl/6 mice on chow and HFD. Weights were collected at 16 weeks before tumor cell injection. (C) Tumor masses from the seven batches for all experimental tumors run on CyTOF. An open square with ‘X’ means that tumor was removed from further analysis due to poor viability in the CyTOF dataset, fewer than 5000 live CD45^+^ cells. (D) Representative gating strategy for identifying live CD45^+^ tumor-infiltrating leukocytes. NA, nucleic acid; marks DNA and RNA in nucleated cells. Cis, cisplatin; used as a membrane exclusion molecule for the viability assay. (E) Sankey plot visualization of CD45^+^ live cells from total raw events collected for each experimental batch.

To mimic an obese environment, both male and female C57Bl/6 wild-type (WT) mice were fed HFD or chow for 10 weeks prior to orthotopic transplantation of cancer cells (Fig. 1A). Higher body weights were observed in the HFD-fed mice compared to the chow-fed mice for males and females (Fig. 1B). Murine cancer cells were then implanted orthotopically and tumors allowed to form while the mice were kept on their respective diets. To compile a model-independent systematic analysis of the tumor immune effects of the obese environment, we investigated five syngeneic cell line tumor models in two cancer types (mammary adenocarcinoma: E0771, TeLi and Wnt1; pancreatic ductal adenocarcinoma: C11 and UN-KC; Table 1). Consistently, across the seven batches, tumors grown in the obese environment displayed larger tumor mass than those grown in non-obese environments (Fig. 1C).

**Table 1.**
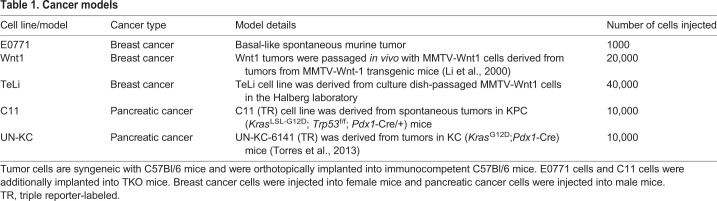
**Cancer models**

Tumors grown in obese or non-obese environments were then dissociated and immunophenotyped with a 36-marker immune-focused panel (Table 2) and analyzed by Helios CyTOF mass cytometry (Fig. 1A). Single-cell data from the five tumor models were collected in seven individually barcoded batches (Fig. 1A), allowing for samples in a single batch to be readily compared. Flow cytometry standard (FCS) files were pre-processed, and live CD45^+^ (also known as PTPRC^+^) cells were gated separately for each batch (Fig. 1A,D). For each experimental cohort, 2.6-13.2 million total raw events were collected (see Table 3 for details). Tumor samples were not enriched for immune cells in advance to avoid experimental bias, and preliminary data indicated that the immune cells comprise ∼5% of the total collected events. With this in mind, the goal was to collect 100,000 to 1 million events per barcoded sample. Because of the lower percentage of target cells, the maximum number of barcodes used for a single batch was limited to 14. The percentage of immune cells per batch ended up ranging from ∼1% to 12% (Table 3). In all tumor models, except TeLi, less than 5% of events collected were gated as live CD45^+^ single cells (Fig. 1E).

**Table 2.**
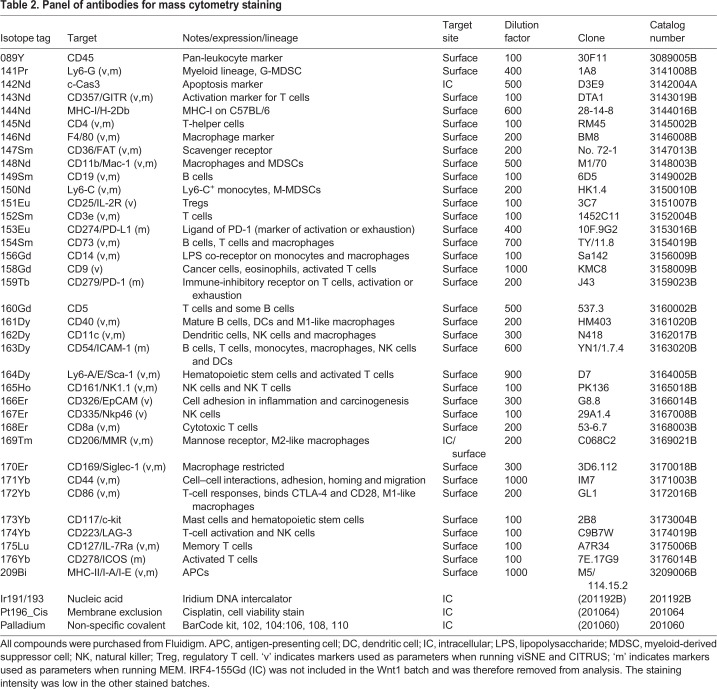
**Panel of antibodies for mass cytometry staining**

**Table 3.**
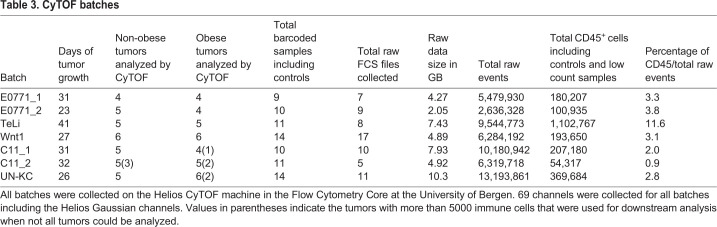
**CyTOF batches**

### Unanchored range-based batch correction enabled successful co-analysis of CyTOF data from multiple barcoded batches

**Fig. 2.**
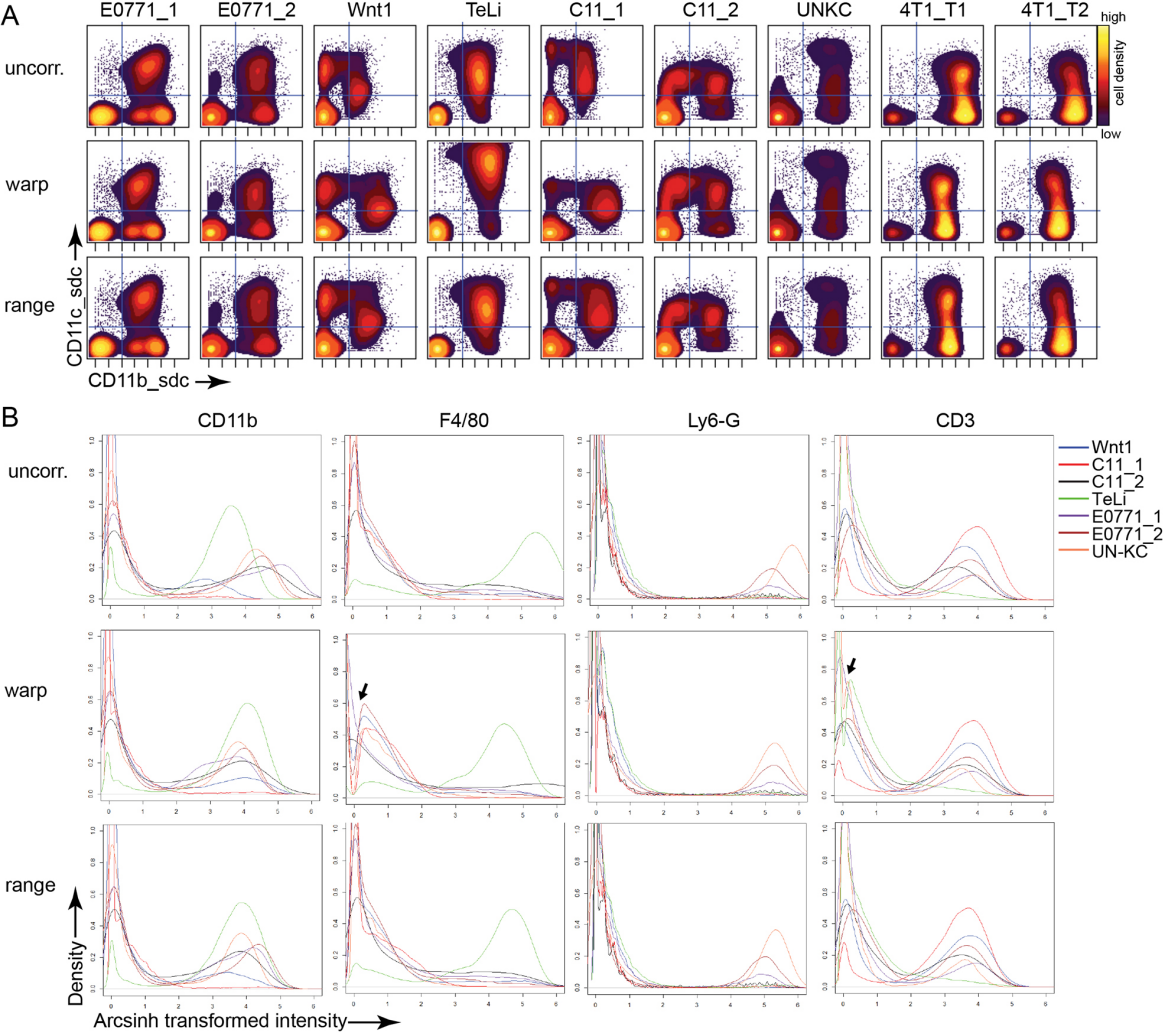
**Batch correction algorithm testing.** All plots were generated from normalized arcsinh-transformed live CD45^+^ cells. Transformed datasets were warp and range corrected, resulting in three datasets, including the uncorrected (uncorr.) dataset. (A) Biaxial contour density plots from the nine testing batches with 18 common markers. The files displayed are the first chow/control sample from each batch. The quadrant gate is shown to assist in visual comparison between plots. (B) Cydar batch correction density plots of four representative markers, showing the third file from each of the seven experimental batches with 36 common markers in total. Black arrows indicate a gap in the density near zero created by the warp correction algorithm.

Streamlined analysis between batches is limited by technical issues such as staining intensity and machine variation (Leipold et al., 2018; Leipold, 2015; Schuyler et al., 2019; Kleinsteuber et al., 2016). To enable streamlined cross-analysis between tumor batches and models, we tested three unanchored batch correction algorithms available through Cydar (Lun et al., 2017). To test the robustness of the batch correction, we combined our seven batches with two additional batches of tumor immune cells from 4T1 syngeneic tumors grown in BALB/c mice with a different cell history and staining panel. We first tested batch correction using warp, quantile and range batch correction approaches on the nine testing batches with 18 shared markers (Fig. 1A, Table 4; Fig. S1). The need for batch correction can be seen in the variable distribution of CD11b (also known as Itgam) and F4/80 (also known as Adgre1) positivity in the uncorrected plots (Fig. 2A; Fig. S1A). Pre-batch correction, the CD11b signal is low for C11_1, Wnt1 and TeLi, and high for both 4T1 batches. The signal intensity becomes more normalized with warp and range batch correction applied. Quantile batch correction, on the other hand, performed poorly and caused an increase in noise and a distortion of the density distribution in the samples, as indicated by the black arrows in Fig. S1A. This distortion of the data is most clearly observed for Ly6-G, for which the need for batch correction was minimal, as seen by the closely aligned peaks in the uncorrected Ly6-G plot. Warp and range correction had similar performance to each other and were therefore further evaluated with the nine testing batches (Fig. 2A; Fig. S1B-D).

**Table 4.**
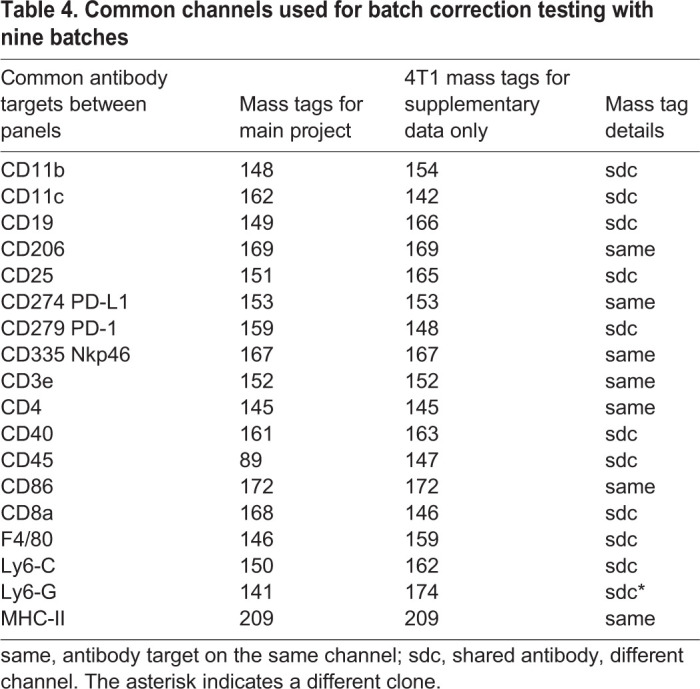
**Common channels used for batch correction testing with nine batches**

Dimensionality reduction was performed with the Cytobank viSNE implementation of BH-tSNE (Kotecha et al., 2010; Amir et al., 2013). Warp- and range-corrected datasets resulted in almost identical viSNE maps when run together (Fig. S1B) and separately with the same seed (Fig. S1C). The marker signal intensity varied somewhat between warp- and range-corrected files run in the same viSNE, but the cell placement on the viSNE map was almost identical (Fig. S1B). This alignment of cell placement can also be seen in the linear regression of the tSNE1 and tSNE2 channels between warp and range (Fig. S1D). Although the warp correction algorithm closely aligns the density peaks (Fig. S1A), it also added distortion artefacts to the data. The warp correction distorted the CD11c (also known as Itgax) signal in the TeLi plot, making the signal higher than in the uncorrected data and higher than in the other batches (Fig. 2A; Fig. S1C, black arrow). The range-corrected plots showed a more consistent maximum intensity for CD11c. The warp correction also resulted in an unexplained bunching artefact for the E0771_2 plot, wherein the cells in the lower left of the map were stacked in a small area (Fig. S1C, pink arrow). To make the final determination between warp and range correction, the 4T1 batches were removed, the seven experimental batches were batch corrected by warp and range methods for 35 markers, and the density plots for key markers were evaluated (Fig. 2B). For several markers, including F4/80 and CD3 (also known as Cd3e), the warp correction created an artificial gap in the data around zero, as indicated by the black arrows in Fig. 2B. Because of this and previously mentioned warping artefacts, Cydar's range correction algorithm was chosen as the batch correction method for the seven experimental batches. With successful batch correction, the data from the five models could then be analyzed in concert and a uniform analysis pipeline was implemented.

### viSNE-based immunotyping revealed diverse myeloid and lymphoid immune infiltrate across the tumor models

**Fig. 3.**
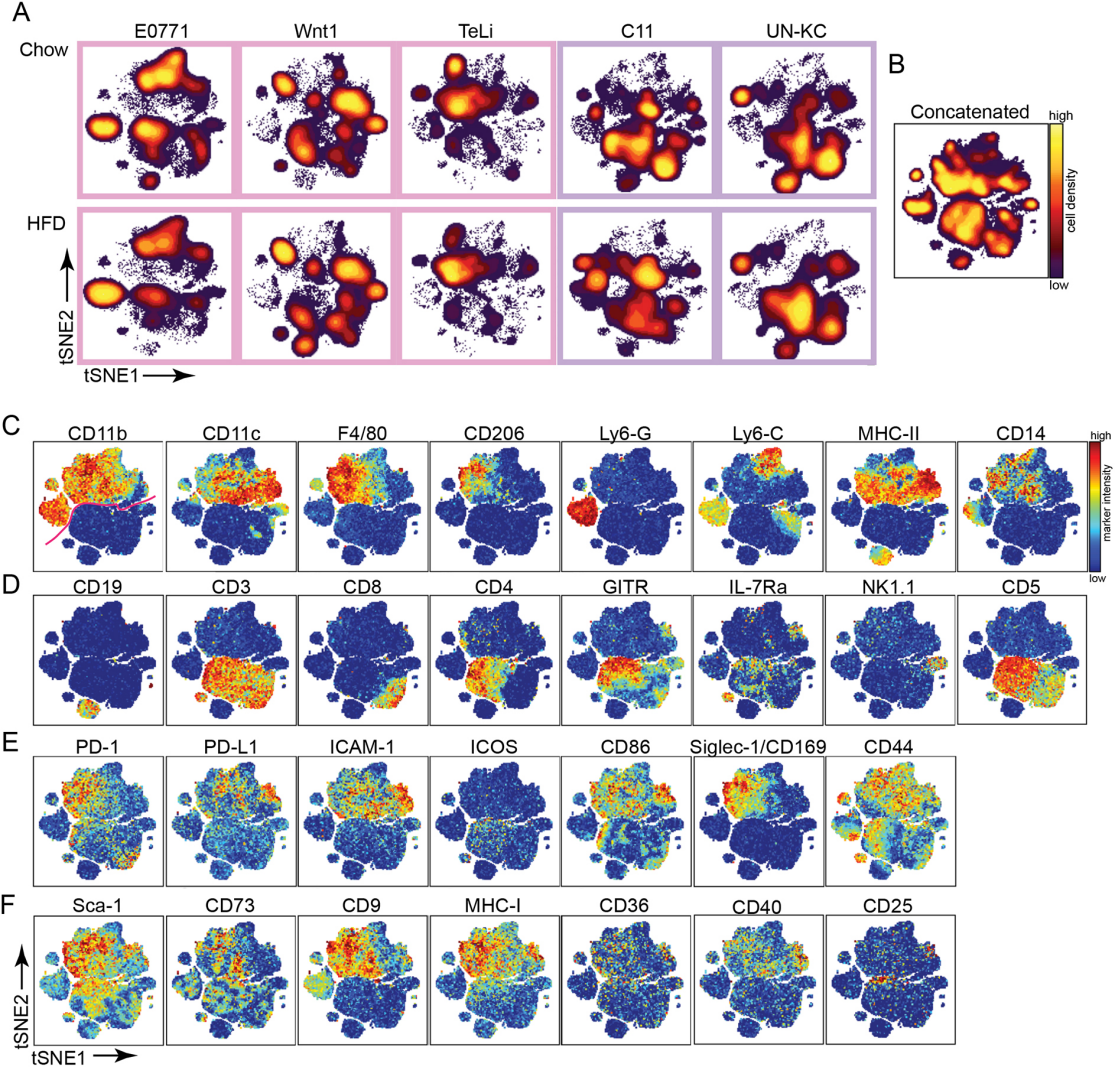
**Immune infiltrate phenotyping using viSNE.** All range-corrected experimental files were run together in the same viSNE run to generate one universal viSNE map. (A) Cell density on viSNE plots for concatenated experimental files for chow and HFD groups in each cohort. E0771 (*n*=9/8), Wnt1 (*n*=6/6), TeLi (*n*=5/5), C11 (*n*=8/3), UN-KC (*n*=5/2). (B-F) The total concatenated data were used to generate viSNE plots (*n*=57). (B) Density plot of total concatenated cells. (C-F) viSNE plots showing marker intensity on a spectrum heat scale. Heat scales are specific to individual markers. (C) Marker heat for key myeloid phenotypic markers. The pink line on the CD11b plot indicates the phenotypic divide between myeloid and lymphoid cells. (D) Marker heat for key lymphocyte phenotypic markers. (E) Marker heat for activation/exhaustion markers. (F) Marker heat for additional phenotyping markers.

Having successfully batch corrected the experimental data, we next moved onto the analysis pipeline, beginning with dimensionality reduction using viSNE (Kotecha et al., 2010; Amir et al., 2013). First, 5206 live CD45^+^ immune cells from each of 57 experimental files and ten control files from the seven batches were run together in a single viSNE analysis with a final Kullback–Leibler (KL) divergence of 4.75. Then, 26 phenotyping markers were used to generate the viSNE map (see Table 2 for details). Cell density was plotted onto the viSNE map to visualize the overall cell distribution and heterogeneity of the tumor immune infiltrate (Fig. 3A,B). The presence of the multiple density ‘islands’ indicates a successful viSNE run and a diverse range of immune cells present across tumor types and diet groups. When assessing density differences between the plots, the largest differences appear to be between tumor models rather than between diet groups (Fig. 3A).

To enable visualization of all the data in one plot, the files were concatenated (Fig. 3B-F). Interrogating the viSNE map by marker intensity revealed the top half (denoted by the pink line in the first plot in Fig. 3C) to be dominated by areas of distinct myeloid marker expression [CD11b, CD11c, F4/80, CD206 (also known as MRC1), Ly6-G, Ly6-C, MHC-II and CD14], indicating a diverse myeloid cell tumor infiltrate. Similarly, the bottom half of the map consists of diverse lymphocyte populations, which can be identified by the marker heats for CD19, CD3, CD8, CD4, GITR (also known as Tnfrsf18), IL-7Ra (also known as IL7R) and NK1.1 (also known as Klrb1c) (Fig. 3D). Cells can be further characterized by looking at the marker expression for activation and exhaustion markers (Fig. 3E) and additional phenotyping markers (Fig. 3F) on the viSNE map.

### Cross-model immunophenotyping of tumor immune cell metaclusters

**Fig. 4.**
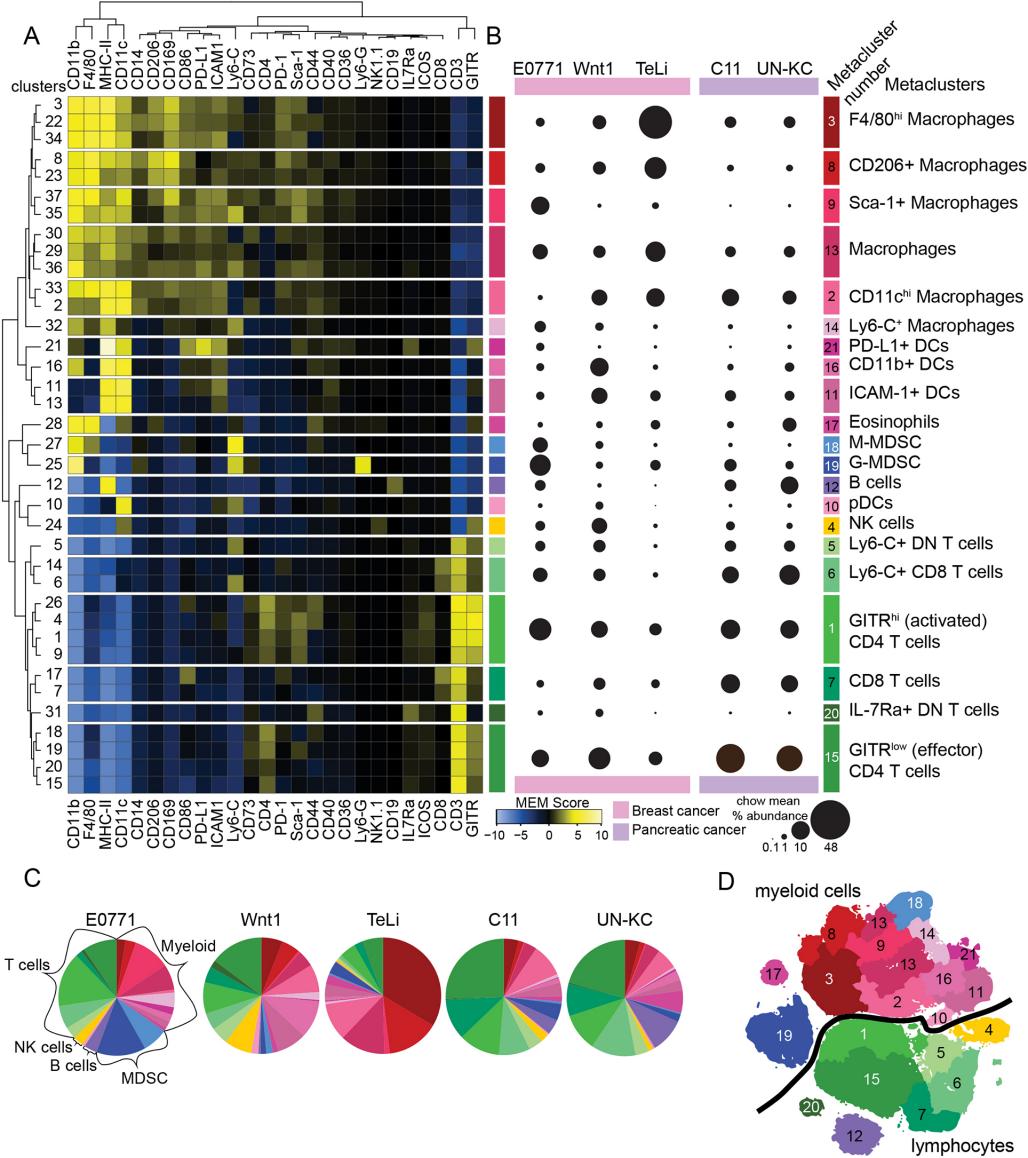
**Immune infiltrate metacluster characterization of murine breast and pancreatic cancers.** (A) Hierarchical clustering of MEM scores for 37 curated clusters and 26 markers. The dendrogram on the left was used to create 21 metaclusters. MEM scores and marker heat were used to label the metaclusters (labels in B). *n*=57: E0771 (*n*=9/8), Wnt1 (*n*=6/6), TeLi (*n*=5/5), C11 (*n*=8/3), UN-KC (*n*=5/2). B and C show mean values for immune infiltrate metaclusters form chow-fed mice; E0771 (*n*=9), Wnt1 (*n*=6), TeLi (*n*=5), C11 (*n*=8), UN-KC (*n*=5). (B) Bubble graph using area to show the mean percentage abundance out of the total CD45^+^ cells for each metacluster for immune infiltrate of each chow-fed non-obese tumor type. (C) Pie charts showing mean percentage abundance of the 21 metaclusters across the five models for the chow tumors. (D) Annotated viSNE map of concatenated data, showing the metaclusters by number. The black line indicates the divide between myeloid and lymphoid lineage cells/clusters (*n*=57).

We next wanted to identify and characterize the different immune cell subsets to compare cell type abundances between tumor models. To perform this analysis with minimal bias and without manual gating, we performed a series of clustering and curating steps to establish 21 biologically meaningful metaclusters. tSNE1 and tSNE2 were used as the input parameters for SPADE-based k-means clustering so that the results of the dimensionality reduction would be preserved in the clusters (Qiu et al., 2011). We used a k of 40 for this first step to capture the immune diversity while reducing the possibility of under-clustering. The 40 clusters were then manually curated to reduce obvious over-clustering by combining clusters within small islands for G-MDSC and NK cells, resulting in a total of 37 clusters. To phenotypically characterize the 37 clusters and to hierarchically cluster them into metaclusters, we next used 26 markers to generate the marker enrichment modeling (MEM) scores and to perform hierarchical clustering of the SPADE clusters and markers based on their MEM scores (Fig. 4A, Table 2) (https://github.com/cytolab/mem). MEM provides insight into how a cluster is positively (yellow) or negatively (blue) enriched for a specific marker compared to the other cells from the other clusters (Diggins et al., 2017). The cluster dendrogram (left side of Fig. 4A) was used to create 21 metaclusters (Fig. 4B). The MEM heat map and scores, median heat map (Fig. S2A) and viSNE plots (Fig. 3B-E) were subsequently used to identify and label the 21 metaclusters. Automated clustering instead of manual gating within the CD45^+^ cells means that no cells were excluded or double counted and user bias was minimized. The multistep clustering pipeline (k-means clustering, followed by manual curation and then dendrogram metaclustering) ensured accurate placement for each cell while minimizing over- and under-clustering. CD11b, F4/80, MHC-II, CD11c, Ly6-C, CD3 and GITR had the largest contribution to the MEM hierarchical clustering, as determined by the marker dendrogram at the top of Fig. 4A. These are bright markers present on many cells with large expression differences between the clusters. This analysis pipeline allowed us to confidently identify six macrophage, six T-cell, four DC and two MDSC metaclusters, in addition to B cells, NK cells and eosinophils. All samples were represented in all 21 of the metaclusters. An overview of the mean percentage metacluster abundance for the non-obese group in each model is shown in the bubble graph in Fig. 4B, and immune cell populations from the chow control groups in the different tumors are displayed as pie charts in Fig. 4C. The concatenated metacluster data were plotted onto the viSNE map for visualization (Fig. 4D).

Of the six macrophage metaclusters, MC8 was M2-like and MC2 was M1-like in phenotype. The CD206^+^ macrophages in MC8 were also enriched for CD14 and CD169 and had decreased expression of MHC-II. The M1-like CD11c^+^ macrophages (MC2) were more abundant in pancreatic cancer models and in the Wnt1 breast cancer model. The M2-like macrophages (MC8) were more abundant in the E0771 and TeLi models (Fig. 4B). The other macrophage subsets are discussed below. The E0771 tumors had the largest abundance of Sca-1^+^ (also known as Atxn1^+^) macrophages (MC9) with ∼10%, whereas they were minimally observed in the other models. MC3 macrophages were especially enriched for F4/80, whereas MC13 macrophages had no strong identifying markers. TeLi tumor infiltrate was dominated by macrophages, specifically from MC3 F4/80^hi^ macrophages, with 73% of TeLi chow immune infiltrate falling into the six-macrophage metaclusters (Fig. 4B,C). The other tumor models had a more diverse immune infiltrate makeup (Fig. 4B,C). MC14 was a small subset of Ly6-C^+^ macrophages, with the greatest abundance in the E0771 and Wnt1 models. The breast cancer tumor immune infiltrate was dominated by myeloid cells, whereas pancreatic cancer tumor immune infiltrate was dominated by T cells (Fig. S2B,C).

Of the six T-cell metaclusters, two were CD8^+^, two were CD4^+^ and two were double negative (DN). The CD8^+^ T-cell metaclusters MC6 and MC7 were touching on the viSNE map and quite similar in phenotype (Fig. 4A,B,D). MC6 contained Ly6-C^+^ and Sca-1^+^ CD8 T cells. MC6 and MC7 were most abundant in the pancreatic tumor models, with C11 average chow infiltrate at 9% and 11% and UNKC chow infiltrate at 12% and 9%, respectively (Fig. 4). Both CD8 T-cell subsets were present across tumor models and expressed similar levels of PD-1 (Fig. 3E). The CD4 T cells fell into an activated GITR^hi^ subset (MC1) and an effector subset that was GITR^low^ (MC15). The DN T-cell metaclusters were phenotypically distinct on the viSNE map and in the MEM heatmap, with MC5 consisting of L6-C^+^ DN T cells and MC20 consisting of IL-7Ra^+^ DN T cells (Fig. 4A,B,D).

Four DC metaclusters were identified in the five tumor models. DCs in the four metaclusters were CD11c^+^, F4/80^−^, CD206^−^ and CD14^−^. MC21 was composed of PD-L1^+^ DCs that were also enriched in MHC-II, CD86, ICAM-1, IL7Ra and GITR (Fig. 4A; Fig. S2A) (ten Broeke et al., 2013). MC21 was a small metacluster with less than 1% abundance in most cohorts and the largest abundance in E0771, which was less than 4% (Fig. 4B). Plasmacytoid DCs (pDCs) (MC10) were the least abundant of the DC metaclusters across all models, less than 2% for Wnt1 tumors and less than 1% for all other models (Fig. 4B,C). An ICAM-1^+^ DC metacluster (MC11) was the second-largest DC metacluster for the Wnt1 model and the largest metacluster for the other four models. Wnt1 had the most DCs of the tumor models, with DCs primarily falling into MC16 and MC11. MC16 was composed of CD11b^+^ DCs and was the most abundant metacluster for the Wnt1 model, with ∼8% for chow tumors.

MDSCs were subsetted into two metaclusters, G-MDSC (also known as PMN-MDSC) in MC19 and M-MDSC in MC18. Both cell types are CD11b^+^; G-MDSCs are Ly6-G^+^ and Ly6-C^low^, while M-MDSCs are Ly6-C^+^ and Ly6-G^−^ (Bronte et al., 2016). MDSCs are known suppressive cells, with G-MDSCs resembling granulocytes and M-MDSCs resembling monocytes. The G-MDSCs in MC19 were particularly abundant in the E0771 chow tumors (Fig. 4B,C).

For the non-obese groups, this unbiased analysis highlights a remarkable heterogeneity between the syngeneic models. The overall myeloid cell abundance was 50% or greater for the breast cancer models and less than 40% for the pancreatic cancer models. In the pancreatic tumor models, over 50% of the infiltrate was T cells (Fig. 4C; Fig. S2C). A comparison of breast and pancreatic cancer immune infiltrate for the non-obese groups revealed multiple significant differences in metacluster abundance (Fig. S2B). However, a limitation is that additional variables such as sex, cancer cell line and tumor location have not been controlled for in this analysis.

### The obese microenvironment leads to model-specific alterations in immune cell populations

**Fig. 5.**
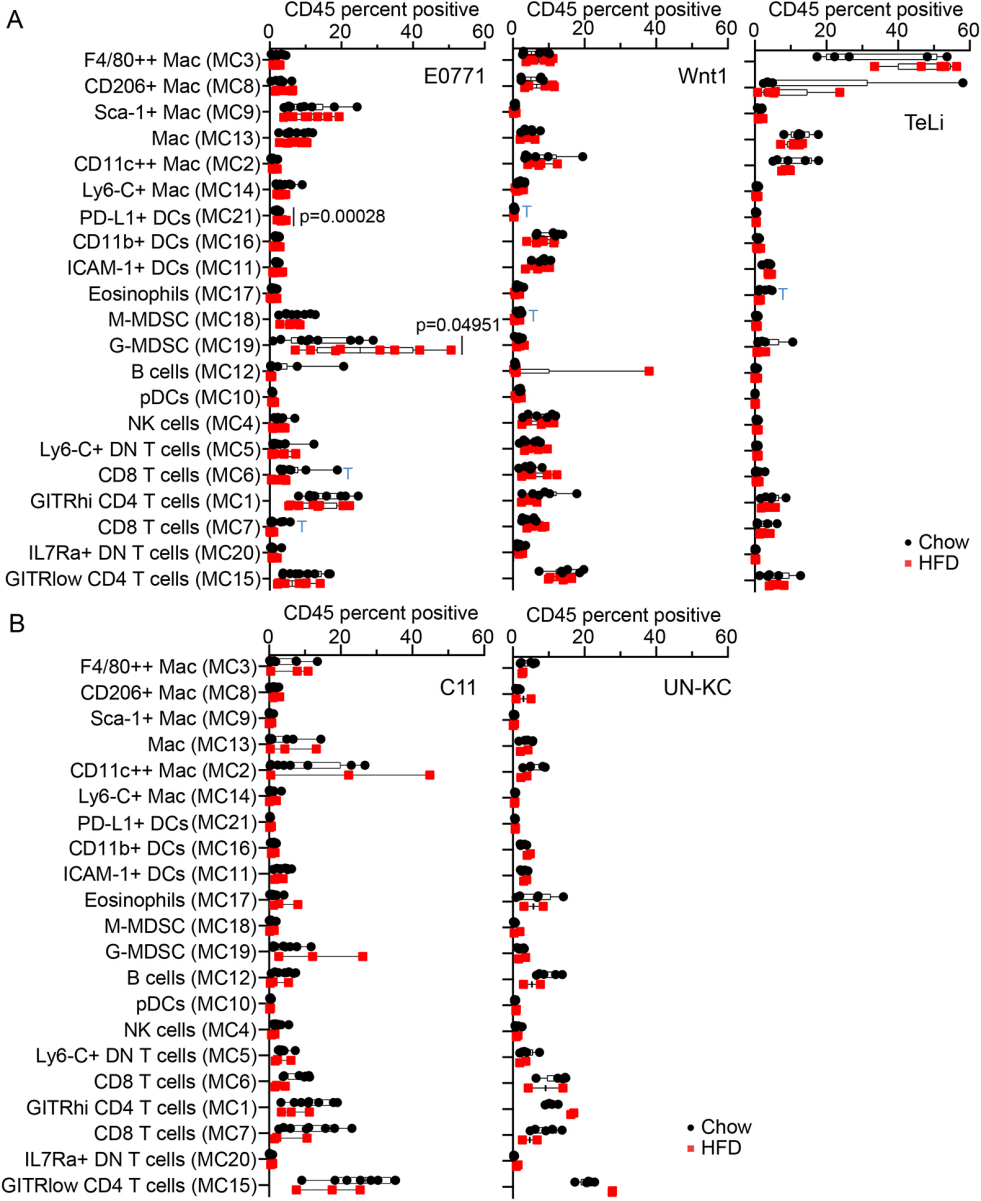
**Analysis and quantification of metacluster abundance differences between chow and HFD.** (A,B) Box and whisker plots with all data points shown (mean, minimum to maximum). (A) Box and whisker plots comparing chow and HFD metaclusters for the breast cancer cohorts. Unpaired Student's *t*-tests were not adjusted, with s.d. assumed between chow and HFD for individual metacluster comparisons. Significant *P*-values are shown. The blue ‘T’s indicate *P*-values less than 0.07 that are trending towards significance. E0771 (*n*=9/8), Wnt1 (*n*=6/6), TeLi (*n*=5/5). (B) Box and whisker plots for pancreatic cancer cohorts. There were too few HFD tumors with live immune cells so statistics could not be performed for those cohorts. C11 (*n*=8/3), UN-KC (*n*=5/2).

Having defined 21 phenotypically relevant metaclusters, we next asked whether an obese environment was associated with differential abundance in immune cell types. Metacluster percentages out of total tumor-infiltrating immune cells were plotted for chow and HFD tumors for each model (Fig. 5A,B).

Surprisingly, the two Wnt1-driven mammary tumor models (Wnt1 and TeLi) displayed no significant differences between chow and HFD in tumor immune infiltrate (Fig. 5A). However, in the E0771 breast cancer model, we observed significant differences in a small PD-L1^+^ DC metacluster (MC21) and the large G-MDSC metacluster (MC19), both showing increased abundance in HFD (Fig. 5A). The E0771 tumors from obese mice contained the highest percentage of G-MDSCs, which was significantly more than the tumors from non-obese mice (Fig. 5A). Both CD8 T-cell metaclusters (MC6 and MC7) were trending towards a decrease in HFD (Fig. 5A). In the pancreatic cancer models, the obese tumor microenvironment did not lead to any major alterations in the tumor immune infiltrate.

### DIO led to an increased abundance of G-MDSCs and a decrease in CD8 T cells in the E0771 triple-negative breast cancer model

**Fig. 6.**
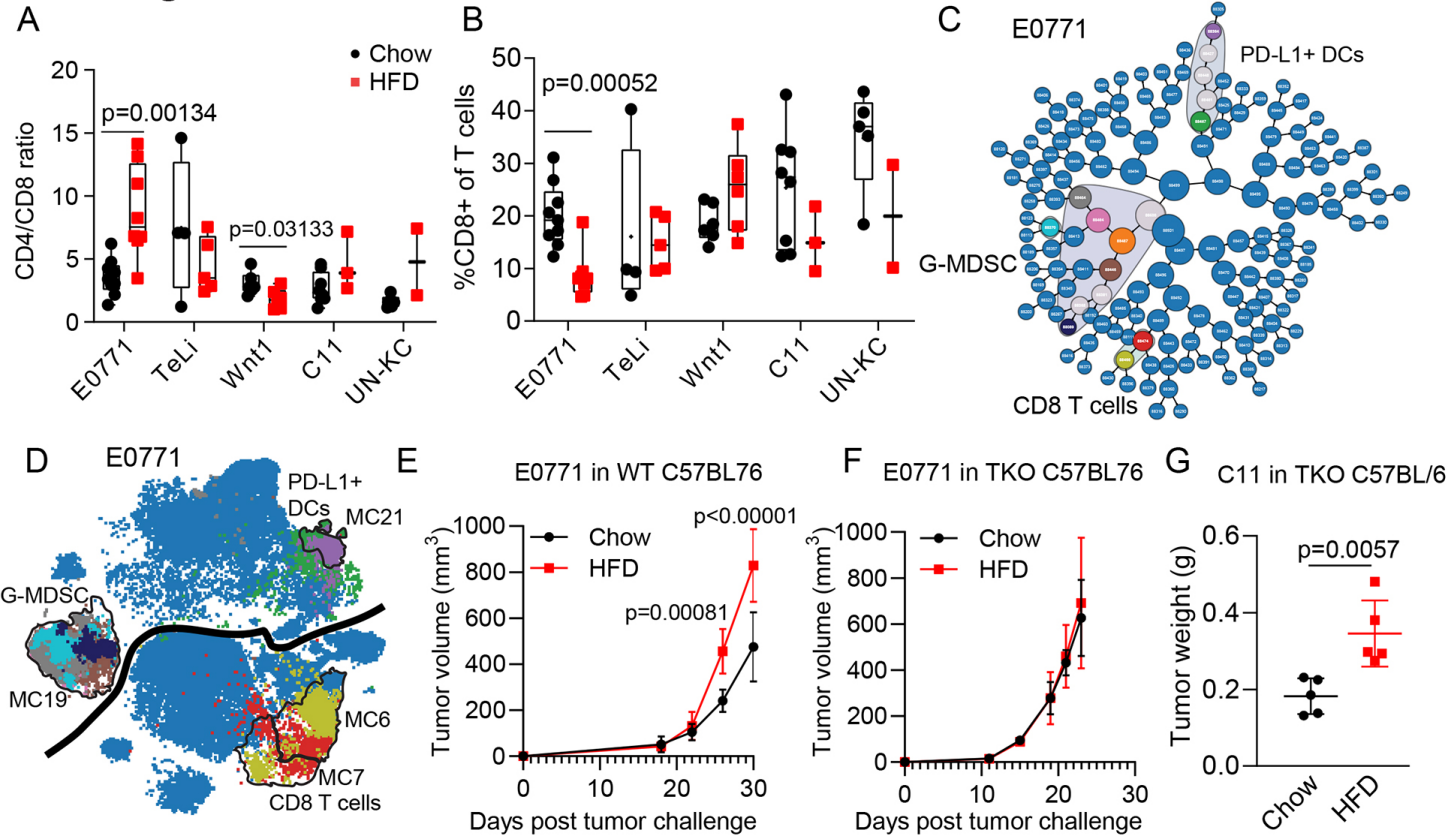
**CD8 T cells were decreased in the HFD DIO E0771 model of triple-negative breast cancer, and tumor growth advantage was lost when the T-cell compartment was lost in the TKO model.** (A,B) Box and whisker plots with all data points shown (mean, minimum to maximum). Unpaired Student's *t*-tests were not adjusted, with s.d. assumed between chow and HFD for individual cell subsets. E0771 (*n*=9/8), Wnt1 (*n*=6/6), TeLi (*n*=5/5), C11 (*n*=8/3), UN-KC (*n*=5/2). (A) CD4/CD8 ratio for each tumor model. (B) Percentage of CD8 T cells of total T cells. (C) CITRUS SAM results for E0771 model. CITRUS clusters that are significantly different between chow and HFD are not blue and are circled with a gray background (*n*=9/8). (D) Selected significant CITRUS clusters were plotted back onto the viSNE map. Plotted clusters are color coded to match the CITRUS plot in C. viSNE data shown are concatenated data for the ten CITRUS clusters and total cell numbers for the E0771 model. The black line indicates the divide between myeloid and lymphoid lineage cells/clusters. (E,F) Tumor growth volume over time for E0771 WT (*n*=4/4; E) and E0771 TKO (*n*=5/5; F) cohorts (mean±s.d.). Unpaired Student's *t*-tests were not adjusted, with s.d. assumed. (G) Final tumor masses for C11 tumors grown in TKO mice, showing that the HFD tumor growth difference remains (*n*=5/5). The appropriate comparison is to the C11 tumor masses for C11_1 and C11_2 batches shown in Fig. 1C. Data are graphed as scatter dot plots (mean±s.d.). Unpaired Student's *t*-test, with s.d. assumed.

To further explore the connection between the obese microenvironment and immune infiltrate in the E0771 model, we examined the ratio between CD4 and CD8 T cells. This ratio has been used in peripheral blood and tumor tissue as a measure of immune health (Das et al., 2018). In breast cancer, an elevated CD4/CD8 ratio has been associated with tumor progression and poor survival (Yang et al., 2017; Wang et al., 2017). Here, we found that the CD4/CD8 ratio was higher in tumors that evolved in obese compared to non-obese mice in the E0771 model (Fig. 6A). Further, the CD8 T-cell percentage out of the total T cells was significantly decreased in HFD tumors in the E0771 model (Fig. 6B). We did not detect differences for the CD4 T-cell population in the E0771 model (Fig. S3A). For E0771, the total T cells out of the CD45^+^ cells were trending towards a decrease in HFD but the results were not significant (Fig. S3B).

To independently validate the E0771 findings, we next ran a CITRUS analysis on the E0771 cohort (Bruggner et al., 2014). Consistently, the CITRUS SAM model found significant cell abundance differences for the three groups described above: G-MDSCs, CD8 T cells and MHC-II^hi^ DCs (Fig. 6C,D). A subset of the significant clusters was mapped onto the viSNE map for cell type identification and visualization (Fig. 6D). The abundance differences were in the same direction as identified in the metaclusters in Fig. 5A, with G-MDSC and MHC-II^hi^ DCs increasing in HFD and CD8 T cells decreasing in HFD (Fig. S3C). G-MDSCs have been reported to inhibit CD8 T-cell function and proliferation (Youn and Gabrilovich, 2010). We thus hypothesized that the increased G-MDSC population was inhibiting CD8 T-cell effector function and proliferation, resulting in larger tumors in the obese E0771 model.

To functionally test this, we implanted E0771 cancer cells into the mammary gland of C57Bl/6 mice deficient in T, B and NK cells [*Rag2*^−/−^::*Cd47*^−/−^::*Il2rg*^−/−^; triple knockout (TKO)] and compared the tumor growth to tumors in WT C57Bl/6 mice. Overall, the E0771 tumors grew faster in the TKO model. However, interestingly, the tumor growth advantage observed in the obese environment of WT mice was abrogated in the immune-deficient environment in the TKO mice (Fig. 6E,F). Consistent with our immune profiling, this suggests that E0771 cancer cells that evolve in the obese environment have the ability to attract G-MDSCs to help overcome T-cell cytotoxicity. This renders tumor growth independent of T-cell infiltration in the obese state. In the non-obese state, however, E0771 tumor growth is affected by cytotoxic T cells, and tumor growth is therefore enhanced in the non-obese TKO mice. In addition to the E0771 model, we also performed the TKO experiment in the C11 pancreas tumor model. Our immunotyping of this tumor did not suggest any deregulated immune populations in HFD (Fig. 5B). Consistently, the C11 tumor growth advantage in the obese environment was sustained in the TKO model (Fig. 6G). Combined, this demonstrates that, in the E0771 model, tumor evolution in obese environments is linked to a functional differential immune interaction and that such interaction is highly model dependent.

## DISCUSSION

Here, we compared the tumor-infiltrating immune cell populations from seven unanchored batch-corrected Helios CyTOF runs collected from two different tumor types adapted to obese and non-obese environments. The batches were then combined to form five tumor models for in-depth immunotype analysis.

Although anchored batch correction is the new gold standard for batch correction methods, there are many datasets that do not have this luxury. Our unanchored batch correction implementation here enabled the implementation of a single comprehensive analysis pipeline and provides a path forward for the streamlined analysis of other unanchored multi-batch mass cytometry datasets. Being able to analyze datasets together provides a great advantage over analyzing them in parallel. Batch-corrected datasets can be gated, clustered, visualized and statistically analyzed in unison, making for stronger conclusions. Application of unanchored batch correction could allow for the meaningful re-analysis of previously collected datasets that may have been set aside due to batch effects and lack of anchor or reference samples.

The implementation of automated clustering approaches over manual gating have introduced a shift in cell subset classification rendering clustering less reliant on the +/− classification system traditionally used for characterizing cell types (Misharin et al., 2013; Zaynagetdinov et al., 2013). Cluster subsetting herein was performed with dimensionality reduction and a multistep clustering approach that minimized bias due to a lack of manual gating. For example, CD11b^+^ DCs (seen in MC16) have typically been described as F4/80 positive or negative (Misharin et al., 2013), but because we used F4/80 to classify cells, F4/80^+^ cells are separated from F4/80^–^ cells. Here, F4/80^+^ cells were classified as macrophages, eosinophils or M-MDSCs, and all of the DC subsets were F4/80^−^.

Macrophages and DCs form a complex family of myeloid cells with overlapping functions and phenotypes (Misharin et al., 2013). Their highly plastic nature and tissue-specific phenotypes make identification difficult (Leopold Wager and Wormley, 2014). We chose to name the metaclusters with the most likely cell type based on standard marker classifications and by the marker that most distinguished them from the other metaclusters of that cell type. Additionally, we included a wide range of phenotyping data in the figures to act as a resource for others to identify cell types of interest regardless of the name used to define them. This study identified multiple tumor-infiltrating macrophage and DC subsets.

Macrophages, identified by high expression of CD11b, F4/80 and MHC-II, fell into six metaclusters representing macrophages of differing phenotypes and function. CD206 expression is associated with an M2 or pro-tumor phenotype (Haque et al., 2019; Nawaz et al., 2017). CD11c^hi^ macrophages (MC2) were phenotypically similar to an M1, known to be an anti-tumor phenotype, macrophage (Zhu et al., 2017; Gautier et al., 2012; Noy and Pollard, 2014). The CD11c^hi^ macrophages were also high for MHC-II, indicating an activated state (ten Broeke et al., 2013). MC14 was composed of Ly6-C^+^ macrophages, which have been described in the literature as playing a detrimental role in multiple disease states (Gibbons et al., 2011; Kimball et al., 2018). The Ly6-C^+^ macrophages may also represent newly infiltrating monocytes, which may further differentiate into other macrophage phenotypes (Noy and Pollard, 2014; Movahedi et al., 2010; Rahman et al., 2017). Sca-1^+^ macrophages (MC9) were additionally enriched for ICAM-1 and PD-L1. ICAM-1 has been reported to have anti-tumor effects, whereas PD-L1 is associated with T-cell inhibition with pro-tumor effects (Yang et al., 2015; Han et al., 2020; Patsoukis et al., 2012). Sca-1 expression is associated with stemness and a self-renewing state (Walasek et al., 2013), making this macrophage phenotype particularly complex. These six metaclusters reveal the complexity of tumor-associated macrophages and quantify their contributions to the tumor microenvironment.

It is interesting to note that the Wnt1 and TeLi immunotypes are so different, with TeLi infiltrate being dominated by macrophages. These models only differ by passaging method; Wnt1 cells are passaged *in vivo*, whereas TeLi cells are Wnt1-derived cells that have been established and passaged *in vitro*.

Surprisingly, this extensive and unbiased analysis did not reveal any differences in tumor-infiltrating macrophage populations between either breast or pancreas tumors grown in obese and non-obese environments. This is in contrast to literature describing the influx of macrophages in obese adipose tissue (Weisberg et al., 2003). There is an assumption that increased macrophages in adipose tissue will correspond to increased macrophages in tumors. This has not been well documented and direct comparisons with the literature are difficult to make. Tumor-infiltrating macrophage increases are often determined by changes in gene or protein expression and not at the single-cell level (Cranford et al., 2019). The inclusion of different tumor models and their knockout counterpart analyses also make it difficult to compare macrophage abundance between obese and non-obese tumor microenvironments (Incio et al., 2016b). It is possible that tumor-infiltrating macrophage differences are not in abundance and that a more macrophage-focused panel might reveal macrophage phenotypic differences between the obese and non-obese setting.

DCs are major antigen presenters and good targets for anti-tumor immunity therapy (Wculek et al., 2020). This mass cytometry study was not specifically designed to subset DCs, but the analysis pipeline still managed to identify and characterize four DC metaclusters with distinct phenotypes. The MC21 DCs are characterized by high PD-L1 positivity, indicating that this subset is T-cell suppressive (Oh et al., 2020). Although the shift was small, MC21 was significantly increased in the obese E0771 group. The CD11b^+^ DCs found in MC16 are likely to be conventional cDC2 dendritic cells and are strong activators of CD4 T cells (Binnewies et al., 2019). pDCs (MC10) are associated with tumor aggressiveness and poor prognosis (Wylie et al., 2019). Although they were present in all groups, there was no measurable difference in abundance between the different groups, indicating that pDCs do not play a major role in obesity-associated cancer. The addition of CD103 to the panel would also enable the identification of cDC1 subsets, which are important for activating CD8 T cells and play a large role in anti-tumor immunity (Wylie et al., 2019).

The published research on the cancer-obesity link is sizable and growing. There are many different models and experimental designs in use. One key feature of our experimental design is the live cryopreservation of the tumor cells. This approach depletes the neutrophils, enabling a definitive identification and characterization of G-MDSCs (Graham-Pole et al., 1977; Kotsakis et al., 2012). Neutrophils and G-MDSCs are almost phenotypically indistinguishable by fluorescence flow and mass cytometry (Zilio and Serafini, 2016; Zhou et al., 2017). Because neutrophils do not survive the freeze-thaw process, CD11b^+^ Ly6-G^+^ cells in our analysis are G-MDSCs and not neutrophils (Graham-Pole et al., 1977; Kotsakis et al., 2012). Some G-MDSCs were likely lost due to the freeze-thaw process; however, the cell loss remained comparable across samples (Trellakis et al., 2013).

The multistep clustering pipeline provides useful insight into the phenotypic relatedness of these cell types. Owing to differing Ly6-G expression, G-MDSCs (MC19) and M-MDSCs (MC18) are quite distant on the viSNE map. The strong Ly6-G staining on the G-MDSC population contributed to those cells being placed in a separate viSNE island for that metacluster. The similarity of expression for the other markers (mainly CD11b and Ly6-C) resulted in the MDSC metaclusters being very close in the hierarchical clustering. With the data available here, it would be a mistake to combine the MDSC subsets into a single metacluster. The metacluster spacing on the viSNE map makes it clear that they are distinct populations of cells, which is consistent with the literature; however, a less-stringent metaclustering using the dendrogram would have resulted in a single MDSC metacluster, which would have been far less informative and counter to the cell spacing on the viSNE map and the literature. Although we attempted to minimize bias in this analysis pipeline, expert knowledge was still crucial for correctly subsetting these cell types. Here, we found that the G-MDSCs were increased in abundance in the obese E0771 group, meaning that for the E0771 model, there are two distinct T-cell-suppressive cell subsets.

T cells, specifically CD8 T cells, are major players in tumor immunity. Although tumor-infiltrating CD8 T cells, often referred to as TILs, are the most-studied T-cell type in cancer, CD4 and DN T cells are both commonly found in the tumor microenvironment. T cells play various roles in the tumor microenvironment. Here, we found two phenotypically distinct and perhaps functionally distinct CD4 T-cell subsets. The GITR^hi^ CD4 T cells in MC1 were also enriched for Sca-1. Both of these markers indicate an activated phenotype (Whitmire et al., 2009; Nocentini and Riccardi, 2009). GITR is also high on regulatory T cells (Tregs), but there were very few CD25^+^ cells in this metacluster so it is unlikely that there were many Tregs in MC1. Low GITR indicates naïve or effector T cells, which characterize the second CD4 T-cell metacluster, MC15. It is notable here that two distinct DN T-cell populations were identified. The two DN T-cell metaclusters are phenotypically distinct and far apart on the viSNE map. The Ly6-C^+^ DN T cells (MC5) were near the NK cells between the CD4 and CD8 T cells, while the Il-7Ra^+^ DN T cells (MC20) are in a separate island on the far side of the map. DN T cells are known suppressor cells and have been shown to suppress cancer cell growth in culture (Lu et al., 2019; Young et al., 2003).

CD8 T cells are the primary tumor-cytotoxic lymphocyte (Martínez-Lostao et al., 2015). CD8 T cells in cancer are often exhausted and no longer capable of cancer cell killing. Checkpoint blockade immunotherapy (CBI), such as anti-CTLA-4 and anti-PD-1/PD-L1, works to reactivate the CD8 T cells and can lead to better patient outcomes in multiple cancer types (Christofi et al., 2019). CBI only works in a small subset of patients though and it is not clear why (Zugazagoitia et al., 2016). Understanding the CD8 T-cell contribution to the tumor microenvironment is key to taking full advantage of checkpoint blockade therapies. Previous reports have suggested that obese patients tend to have worse responses to traditional cancer therapies but do better with immunotherapy than non-obese patients (Wang et al., 2019). It is not clear why obese patients respond so well to checkpoint blockade immunotherapies, but it may be due to the existing immune dysregulation and chronic low-grade inflammation in obese patients (Naik et al., 2019). It has been shown that patients with lower CD8 expression in the tumor microenvironment tend to have worse outcomes in response to traditional therapies (Mahmoud et al., 2012; Liu et al., 2012). It is not clear how intratumoral CD8 levels relate to CBI success or to obese patient outcome. However, a recent paper by Ringel et al. further examines the connection between T-cell function and cancer in the obese setting using the murine colorectal adenocarcinoma cell line, MC38 (Ringel et al., 2020). Similar to the E0771 results presented here, MC38 tumors developed in obese mice displayed decreased CD8 and increased MDSC infiltration. Mechanistically, cellular adaptations to the obese environment resulted in differential fatty acid uptake between cancer and CD8 cells, leading to impaired CD8 infiltration and function. Further, depletion of CD8 T cells using an inhibitory antibody reversed obesity-induced growth rates. This is consistent with our findings using the E0771 TKO model. They also examined the E0771 orthotopic tumors; however, they did not see differences in CD8 T-cell or MDSC abundance between chow and HFD. These conflicting results may be due to the large difference in E0771 cell injection numbers [1000 herein compared to 200,000 cells by Ringel et al. (2020)].

Here, we were able to provide an in-depth characterization of multiple T-cell subsets, which could be valuable for future studies in determining the therapeutic value of checkpoint blockade therapies across patients and cancer types. The two CD8 T-cell subsets we found were quite similar in phenotype and both metaclusters were decreased in the obese E0771 group. This fits well with the increased abundance of two T-cell-suppressive cell types, G-MDSCs and PD-L1^+^ DCs.

Although DIO led to tumor immune infiltrate changes in the E0771 model, it did not have an effect across all models. Despite the systemic nature of the obese phenotype, our results suggest that the interplay between obesity and tumor immune infiltrate is very cancer subtype specific. Different treatment approaches might be needed depending on the cancer subtype's ability to alter the tumor immune infiltrate in the obese setting. It is possible that there were detectable obesity-dependent immune changes outside this 36-marker CyTOF panel. Investigating additional activation/inactivation markers, chemokine receptors and cytokine production could shed light on those changes. But, overall, our broadly defined immune panel performed well, and provided deep and robust tumor immune phenotype across models. High-dimensional positional data such as imaging mass cytometry might further help unravel the obesity effect. Several recent studies have indicated that tumoral cell–cell interactions and local neighborhoods play a role in cancer severity (Jackson et al., 2020; Keren et al., 2018; Schürch et al., 2020), indicating that such spatial information adds phenotypic information compared to cell frequencies obtained with suspension-based mass cytometry. The limited data from the TKO mice suggest that the obese immune system might play a stronger role in breast cancer than in pancreatic cancer, at least at the tumor immune infiltrate level. Several studies support this concept, showing that non-immune cell components contribute strongly to pancreatic cancer prognosis in obesity (Chung et al., 2020; Eibl and Rozengurt, 2019).

Here, we have presented a pipeline for analyzing large mass cytometry datasets collected across multiple time points without anchor samples. This is a powerful approach for analyzing data collected before the use of anchor samples was introduced. The multistep clustering approach allows for a reduction of biases while still allowing for expert input to guide the final metaclusters. The detailed characterization of immune infiltrate across five tumor models provides a valuable resource for planning tumor immunity studies. We found that although cell subsets were conserved across models, subset abundance was highly model specific. The inclusion of tumor immune infiltrate from obese groups provides insight into cancer models that may or may not be relevant for studying immune infiltrate differences in obesity. We propose that the E0771 breast cancer model is a clinically relevant model for assessing immune infiltrate in obesity. G-MDSC and PD-L1^+^ DC suppression of T cells is clinically relevant in regard to patient care and treatment options.

## MATERIALS AND METHODS

### Experimental mouse models

The Norwegian Animal Research Authority approved all animal experiments. Experiments were carried out according to the European Convention for the Protection of Vertebrates Used for Scientific Purposes. The Animal Care and Use Programs at the Faculty of Medicine, University of Bergen is accredited by AAALAC International. Male and female WT C57BL/6J (stock number 000664) mice were purchased from The Jackson Laboratory. TKO (*Rag2*^−/−^::*Cd47*^−/−^::*Il2rg*^−/−^) mice were purchased from The Jackson Laboratory (stock number 025730). C57Bl/6 TKO mice are deficient in T, B and NK cells. Mice were kept in IVC-II cages (SealsafeÒ IVC Blue Line 1284L, Tecniplast) and housed in the laboratory animal facility at the University of Bergen. Up to six mice were housed together and maintained under standard housing conditions at 21±0.5°C, 55±5% humidity and 12 h artificial light-dark cycle. Mice were provided with food and water *ad libitum*.

At 6 weeks of age, mice in the obese cohort were randomly placed on a HFD (60% kcal from fat, 20% from protein, and 20% from carbohydrates, Research Diets, D12492) for 10 weeks, while lean mice were kept on a standard chow diet (7.5% kcal from fat, 17.5% from proteins and 75% from carbohydrates, Special Diet Services RM1, 801151). At 16 weeks of age, mice were weighed (Fig. 1B), and breast cancer cell lines were orthotopically injected into the 4th inguinal mammary fat pad for female mice. At the same time point, pancreatic cancer cell lines were orthotopically injected into the lower body of the pancreas of male mice (Fig. 1A). For pancreatic implantations, hair was removed at the incision area and skin disinfected twice with 70% ethanol, before the pancreas was visualized by making a left flank incision in the epidermis and peritoneum below the ribcage. The spleen, body and tail of the pancreas were then gently extra corporealized by the use of forceps and cotton tips. After injection, the muscle layer was closed by suturing (Vicryl suture 5-0, V388H, Ethicon) and the skin was closed by the use of wound clips (EZ Clips™ 59027). To maintain body temperature, mice were placed under a heating lamp for 20 min post-surgery. For mammary tumors, cells in phosphate buffered saline (PBS) were mixed 1:1 by volume with Matrigel (Corning, 356231) and injected in a total volume of 50 μl for fat pad injections and 30 μl for pancreas injections. Feeding regimens were maintained throughout the experiment. Tumor growth and mouse weight were monitored over time. Breast cancer tumor size was measured by caliper. Pancreatic tumors were monitored and removed based on optical imaging of luciferase expressing cancer cells and careful monitoring of mouse distress based on the grimace scale. The experiment was stopped when the first mouse showed signs of distress. At endpoint, the mice were euthanized by cervical dislocation while anesthetized, and tumors were harvested for weight measurements and downstream analysis (Fig. 1A). See Table 1 for tumor model cell line details and cell injection numbers.

The UN-KC cell line was kindly provided by Dr Surinder Batra (University of Nebraska Medical Center, Omaha, NE, USA). The E0771 cell line was acquired from CH3 BioSystems. The *in vivo* passaged MMTV-Wnt1 cells were kindly provided by Stein-Ove Døskeland, University of Bergen. The TeLi cell line was generated in house by *in vitro* passaging of dissociated cells from the MMTV-Wnt1 cell line injected in the mammary fat pad. The tumor was dissociated using a mouse tumor dissociation kit (Miltenyi Biotec, 130-096-730) according to the manufacturer's instructions. Dissociated MMTV-Wnt1 tumor cells were cultured *in vitro* for 2 months to obtain pure tumor cells now referred to as TeLi. E0771, TeLi, C11 and UN-KC cells were cultured at 37°C, 5% CO_2_ in high-glucose Dulbecco's modified Eagle medium (Sigma-Aldrich, D5671) supplemented with 10% fetal bovine serum (FBS; Sigma-Aldrich, F-7524), 100 U/ml penicillin and 100 μg/ml streptomycin (pen/strep; Sigma-Aldrich, P-0781) and 2 mM L-glutamine (Sigma-Aldrich, G-7513). All cell lines were regularly (approximately every 6 months) tested for mycoplasma contamination. Cell lines were not authenticated.

### Tumor processing and dissociation

Tumors were collected from seven different mouse cohorts. Representative tumors (those with tumor mass closest to the median) collected from those seven cohorts became the data for the seven batches collected on the Helios CyTOF (Table 1). Tumors were collected, weighed, minced and incubated with collagenase II (Sigma-Aldrich, C6885) and DNase I (Sigma-Aldrich, DN25) based on the Leelatian protocol (Leelatian et al., 2017). Changes to incubation were as follows: minced tumors in RPMI-1640 (Sigma-Aldrich, R7388) were incubated with enzymes in capped 15 ml conical tubes in a warm water bath with periodic inversion while waiting for all tumors to be collected. Tumors were then placed in a 37°C incubator and rotated on a Ferris wheel for 1 h. DNase was used throughout the dissociation protocol and was especially important for pancreatic tumors in which free DNA was observed without the continuous use of DNase. Cells were counted with Trypan Blue and a Countess™ Automated Cell Counter (Invitrogen) before freezing. Live cells in 10% FBS, 90% dimethyl sulfoxide were cryopreserved with CoolCell (Sigma-Aldrich, CLS432001) at −80°C overnight before transfer to liquid nitrogen. Tumor weights and cell viability are reported in Table 5.

**Table 5.**
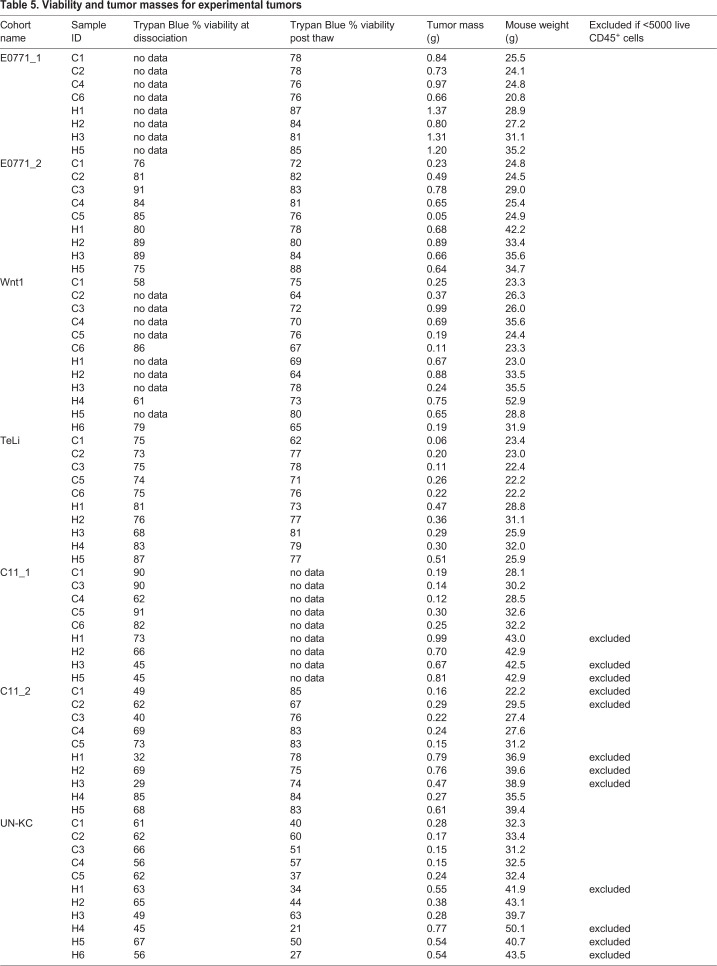
**Viability and tumor masses for experimental tumors**

### Determining the number of samples to barcode and number of cells to collect

Before data collection, it is imperative to know the size of the target population to enable a robust analysis, particularly in heterogeneous populations. Preliminary testing on E0771 tumors suggested that the CD45^+^ target cells were ∼5% of the total events collected; that held roughly true for the barcoded batches (Table 3). The following equations were used to guide the number of samples barcoded together and the time spent at the Helios for collection. The event rate was ∼500 events/s.(1)
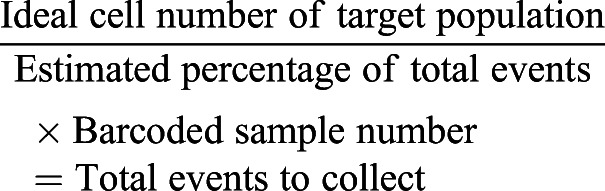
(2)
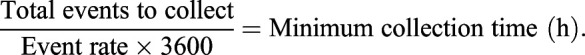


### Cell staining and running on Helios

Cells were thawed in a warm water bath at 37°C, and 1 ml warm DNase-supplemented medium (RPMI-1640 with 10% FBS, 1% pen/step and DNase) was added to each thawed cryovial. Contents were then dumped into labeled 15 ml conical tubes containing 8 ml warm DNase medium. Cryovials were washed with 2 ml warm DNase medium and added to the contents in the corresponding 15 ml conical tube. Cells were then rested for 5 min before being centrifuged at 200 ***g*** for 5 min at room temperature (RT). Cells were again counted with Trypan Blue using the Countess automated cell counter and stained with cisplatin (Fluidigm, 201064) in the DNase medium using the Fluidigm protocol. Cells were kept in warm DNase medium until they were fixed with paraformaldehyde (PFA) at 1.6% final concentration. Following fixation, cells were barcoded using a palladium cell barcoding kit (Fluidigm, 201060) and vendor protocol, with the modification of incubating with barcodes for 45 min instead of 30 min. After barcoding, combined cells were kept in PBS+1% bovine serum albumin (BSA; Sigma-Aldrich, A7030), blocked with anti-CD16/CD32 Fc block (eBoscience, 16-0161-82) and stained with a pre-made cocktail of antibodies shown in Table 2. All antibodies were conjugated and validated by Fluidigm. Following surface staining, cells were permeabilized with 2-3 ml 100% pure cold methanol overnight at −20°C. Cells were vortexed vigorously before and after methanol addition to prevent clumping. The next day, 2 ml PBS was added to dilute methanol. Cells were spun down at 900 ***g*** and methanol/PBS mixture was pipetted off. Cells were washed with PBS again and decanting was performed as usual. Intracellular staining was performed in Dulbecco's PBS (DPBS)+1% BSA for 30 min. After 20 min, DNase and iridium intercalator (Fluidigm, 201192B) were added. DNase was used on fixed and permeabilized cells to reduce clogging on the Helios and cut down on visible clumping when running samples. This was especially important for the pancreatic tumors that seemed to have a high level of cell death and free DNA.

All staining was performed in capped 5 ml FALCON polystyrene tubes (Corning, 352052) on a moving platform or with manual agitation to prevent cells from fully settling. Final washes were all with 3 ml, followed by vortex, centrifugation at RT at 900 ***g*** and decanting: three PBS+1% BSA washes, three PBS washes, three MilliQ washes. Cells were kept in the void volume plus 100 μl of 1× beads in MilliQ water at 4°C until ready to run. Then, 25 µl of cells were removed at a time and added to 2 ml diluted 1× Fluidigm EQ calibration beads in MilliQ water immediately before running on the Helios mass cytometer. Cell concentration was adjusted as needed so cells were running at 300-600 events/s. Cells were run on the Fluidigm Helios mass cytometry machine using a narrow bore injector.

A control sample was included in every batch and was used to manually check for antibody staining and machine performance. Control samples were from two different E0771 tumors that had already been phenotyped and used to test the staining panel. Controls were not used as anchor samples for batch correction because more than one control was used across the batches.

### 4T1 tumor model used for batch correction testing

Control group 4T1 breast cancer tumors from female BALB/c mice from two batches were used to test the robustness of the batch correction algorithms. The 4T1 cell line was purchased from ATCC and cultured in a humidified atmosphere (37°C, 5% CO_2_). The 4T1 cells were cultured in RPMI-1640 medium (Sigma-Aldrich) with 10% FBS, 2 mM L-glutamine and pen/strep. MycoAlert (Lonza, LT07-318) was continuously used to confirm that the cell line was mycoplasma free throughout the study. Female BALB/c mice (Envigo) were fed a standard chow diet. At 4-6 weeks, 2.0×10^5^ 4T1 cells were mixed 1:1 with BD Matrigel Matrix Growth Factor Reduced (BD Biosciences) and injected into the right mammary fat pad.

Upon sacrifice, tumors were harvested and dissociated using a mouse tumor dissociation kit (Miltenyi Biotec, 130-096-730). After dissociation, erythrocytes were lysed with Red Cell Lysis Buffer (Miltenyi Biotec, 130-094-183), according to the manufacturer's protocol.

The dissociated 4T1 tumor cells were stained with cisplatin for viability with RPMI1640 (10% FBS, 0.25 μM cisplatin, RT) for 5 min and fixed with 1.6% PFA (electron microscopy grade, Electron Microscopy Sciences, 15710) before freezing. After 10 min incubation, samples were centrifuged and the supernatant was aspirated before being placed in −80°C freezer until analysis.

The panel contained 18 mass-tagged antibodies with the same target as the seven experimental batches. Many of the same antibody targets were on different channels (marked as ‘sdc’ in Table 4) and one was a different clone. A similar protocol, detailed below, was used for staining and cells were run on the same Helios CyTOF machine.

Prior to staining, samples were thawed at RT and resuspended in cell washing buffer (CWB) [DPBS (Thermo Fisher Scientific, 14040-133) with 1% BSA, 0.02% NaAzide and 0.025% DNaseI (Sigma-Aldrich, DN25-1G)]. Cells were counted using the Countess automated cell counter and ∼3×10^6^ cells per sample were used for barcoding. Counted cells were washed in 1× Maxpar® Barcode Perm Buffer (perm buffer; Fluidigm, 201057) twice, before being resuspended in 1× perm buffer (195 µl). Samples were then mixed with 5 µl barcoding solution (Fluidigm, 201060), or 1 µl (500 µM) Intercalator-103Rh (Fluidigm, 201103A) for the control, and incubated for 30 min at RT. After incubation, cells were washed in CWB twice, then pooled with CWB before washing in Maxpar^®^ Cell Staining Buffer (CSB; Fluidigm, 201068).

Surface antibodies were diluted in CSB, while antibodies targeting intracellular proteins were diluted in CSB/perm (10% 10× perm buffer). Samples were blocked on ice for 10 min using anti-CD16/CD32 (75 µl per 3×10^6^ cells) diluted in CSB. The surface staining antibody cocktail was prepared to total 40 µl per 3 million cells. Samples were incubated with the antibody cocktail for 30 min (RT) before washing in CWB with a 10 min (RT) incubation. Samples were then washed in PBS (2 mM EDTA) and fixed with 200 µl 2% PFA per 3 million cells for 30 min (RT, in the dark). Fixed cells were then washed in CSB/perm twice and blocked with anti-CD16/CD32 in CSB/perm as described above. Samples were incubated with the intracellular staining antibody cocktail for 30 min (RT) before being washed in CWB and subsequently in 2 mM EDTA/PBS. Cellular DNA was stained by incubating the samples in Ir191/193 Intercalator (0.33 µl 500 µM Intercalator-Ir per 2×10^6^ cells, Fluidigm, 201192B) mixed with 2% PFA (1 ml per 20×10^6^ cells) overnight (4°C). The following day, samples were centrifuged and resuspended in CWB with 10 min incubation (RT). Samples were then washed in PBS (2 mM EDTA) and kept on ice until the mass cytometer was ready. Before acquisition, an aliquot of cells was washed in MilliQ water four times (400 ***g***, 5 min, RT). The aliquot was then resuspended in 1× EQ Four Element Calibration Beads solution (Fluidigm, 201078) to a final concentration of ∼1-2×10^6^ cells/ml, and strained through a 40 µm cell strainer. The cells were then acquired on the Helios mass cytometer (Flow Cytometry Core Facility, Department of Clinical Science, University of Bergen). All centrifugation steps were performed with a swing-bucket rotor at 900 ***g*** for 5 min at RT unless otherwise specified.

### Data preprocessing

Raw Helios FCS files were bulk normalized in the Fluidigm CyTOF software version 6.7.1014 using the bead normalization passport EQ-P13H2302_ver2 (https://www.fluidigm.com/binaries/content/documents/fluidigm/resources/helios-user-guide0400250/helios-user-guide0400250/fluidigm%3Afile). The Fluidigm normalization tool was chosen so that all datasets could be normalized to the normalization passport. The ‘Original data’ box was checked and the files were not concatenated at this time. The randomization was set to uniform negative distribution (UND) with linear output values, conversion compatible with FlowJo, and the default time interval normalization of 100 s. Beads were not removed. After normalization, the MATLAB debarcoding tool was used to simultaneously debarcode and concatenate the samples, with the exception of batch C11_1 (Zunder et al., 2015). The C11_1 raw batch files were concatenated using the Fluidigm software before debarcoding because the multiple large files were too much for the MATLAB debarcoding tool to open. Each batch was debarcoded separately with the same filter values set to minimum separation of 0.12 and a maximum Mahalanobis distance of 30.

After debarcoding, the R Premessa package was used to resolve a channel-naming conflict (Fig. 1A) (https://github.com/ParkerICI/premessa). The Wnt1 cohort was not stained for IRF4 so that channel was excluded for all seven batches/cohorts. ‘155Gd_IRF4’ was changed to ‘155Gd’ for all FCS files and the channel was ignored during analysis. The signal was low to negative in all stained cohorts so there was minimal potential for spillover. Premessa was used more extensively in Figs S1-S3 (nine-batch dataset) to merge two very different panels so that the 18 common markers could be analyzed even if they were on different channels or had different naming conventions. The code ‘sdc’, for shared different channel, was created to denote shared antibody targets on different channels between the panels. Figs S1-S3 also required the use of the R package cytofCore to reorder channels in the different panels (https://rdrr.io/github/nolanlab/cytofCore/).

After channel renaming with Premessa, all the newly written FCS files were uploaded to Cytobank to check for panel discrepancies and to gate for live CD45^+^ cells. Gating was performed manually to obtain a population of live CD45^+^ events (Fig. 1D). The four Helios Gaussian parameters (Center, Offset, Width and Residual), Event_length, 140-bead channel and Iridium (193) were gated on versus time using the cleanup strategy recommended by Fluidigm (https://www.fluidigm.com/binaries/content/documents/fluidigm/search/hippo%3Aresultset/approach-to-bivariate-analysis-of-data-acquired-using-the-maxpar-direct-immune-profiling-assay-technical-note/fluidigm%3Afile). Iridium intercalator was used to mark intact cells; cisplatin was used as a membrane exclusion stain to differentiate between live and dead cells. Cleaved caspase3 (c-Cas3) was used to exclude apoptotic cells, and CD45 was used as a pan-leukocyte marker (Fig. 1D).

Gated sample files that contained fewer than 5000 live CD45^+^ singlets were excluded from further analysis. This metric was not pre-established. Because of the low viability of the pancreatic tumors, especially those from HFD, we had to remove samples from all three pancreatic cancer tumor cohorts (Fig. 1C, an open square with ‘X’ means that the tumor was removed from further analysis). Three HFD samples were removed from the C11_1 cohort, two chow and three HFD samples were removed from the C11_2 cohort, and four HFD tumors were removed from the UN-KC cohort. No tumors were removed from the four breast cancer cohorts (Table 1).

Live CD45^+^ cells from the remaining 67 files (including ten controls files) from seven batches were exported to new FCS files and downloaded from Cytobank. The CD45^+^ FCS files were imported to R/RStudio for batch correction using Cydar with ncdfFlow and flowCore used as support packages (Lun et al., 2017; https://rdrr.io/bioc/ncdfFlow/; https://rdrr.io/bioc/flowCore/; https://www.R-project.org; http://www.rstudio.com/). Thirty-five markers were arcsinh transformed with a scale argument/cofactor of 5 before batch correction. c-Cas3 was not batch corrected because it was used only during pregating and was no longer relevant. Cydar offers three batch correction algorithms: warp, range and quantile. All three algorithms were tested without the use of common group/anchor files. Initial batch correction algorithm testing was performed on the chow files from the seven experimental batches and the control group files from two batches of 4T1 tumors from BALB/c mice stained with a different panel and under different conditions to test for algorithm robustness. The seven experimental batches were additionally batch corrected separately from the two testing batches using warp and range correction. Range correction was then chosen as the best algorithm for this use, and the range-corrected data were used throughout the rest of the analysis. New FCS files were created, binding together the original and transformed range-corrected data. The 35 new corrected channels were marked with ‘c_’ as the prefix.

### Analysis pipeline

The range-corrected files were uploaded to Cytobank for analysis. viSNE was run using 26 of the corrected markers (Table 2, Fig. 3). Phenotyping markers were chosen to be included as the viSNE input parameters. Markers that were previously used to define the population, that were low or negative, or markers of activation state were not used to generate the viSNE map. CD5 was also excluded to prevent over-representation of T cells on the map. viSNE settings were 4000 iterations on 348,802 events (5206 events per FCS file) and the default settings for perplexity (30) and theta (0.5) were used. The automatic seed was 21,258,186. The run time was 5.58 h and the final KL divergence was 4.75. SPADE was used for clustering, with tSNE1 and tSNE2 as the clustering channels. This preserved the viSNE dimensionality reduction in the clustering process. All cells from viSNE were put into SPADE with 40 nodes/clusters chosen and the default 10% downsample. Cytobank automatic cluster gating was used to gate the events in all 40 SPADE clusters. Clusters were then manually curated and combined to reduce over-clustering for G-MDSCs and NK cells. The new cluster count was 37 clusters. Cell data for all experimental files were concatenated into one file for each of the 37 clusters using Premessa to concatenate the files. The data were imported into R to generate MEM scores, MEM heat map, median heat map and hierarchical clustering (Fig. 4A; Fig. S2A). Event count numbers for clusters/metaclusters were exported for all FCS files. Excel was used to calculate abundance percentages for each metacluster out of the total CD45^+^ cells. In addition to exporting cluster/metacluster events counts, some manual gating was performed on the viSNE map, and event counts were exported for statistical analysis (Fig. 6A,B; Fig. S3A,B). Total CD3 cells were gated on the viSNE map using CD3 marker intensity, and subsequent CD3 subsets (CD4, CD8 and DN T cells) were biaxially gated using the CD4 and CD8 channels.

CITRUS was run on the E0771 data after viSNE analysis as a confirmative analysis for the metacluster statistics. CITRUS clustering was performed in Cytobank on the same 26 channels as viSNE. There were nine files in the chow group and eight files in the HFD group. All events were sampled with a minimum estimated cluster size of 1% (∼885 events). The Significance Analysis of Microarrays (SAM) association model was used for analysis. Select significant CITRUS clusters were plotted onto the viSNE map for visualization.

### Software sources

The Fluidigm Helios software version 6.7.1014 is available upon request from Fluidigm. Manual gating, viSNE, SPADE and CITRUS were all used on the Cytobank Cellmass enterprise server. The single-cell debarcoding tool is a standalone MATLAB application found on github (https://github.com/nolanlab/single-cell-debarcoder). The R packages can be found on CRAN (https://cran.r-project.org/), Bioconductor (Huber et al., 2015) or github. The devtools package is needed for the installation of github-based R packages (https://cran.r-project.org/web/packages/devtools/index.html). The Cydar (https://www.bioconductor.org/packages/release/bioc/html/cydar.html), ncdfFlow (https://www.bioconductor.org/packages/release/bioc/html/ncdfFlow.html) and flowCore (https://www.bioconductor.org/packages/release/bioc/html/flowCore.html) packages are all available from Bioconductor. Premessa (https://github.com/ParkerICI/premessa), cytofCore (https://github.com/nolanlab/cytofCore) and MEM (v3) (https://github.com/cytolab/mem) can all be found on github.

### Statistics

Abundance percentages were calculated in Excel and transferred to GraphPad Prism to perform statistics. GraphPad Prism 8.4.3 was used to plot the data and to calculate *P*-values using multiple unpaired two-tailed Student's *t*-tests. Consistent standard deviation was not assumed between metaclusters and cell subsets, but was assumed between chow and HFD within the Student's *t*-test comparison. Statistics were not applied for UN-KC and C11 lines owing to the limited sample numbers caused by low cell viability. The two batches for both C11 and E0771 were combined so that large trends would be observed and noise would be minimized.

## Supplementary Material

Supplementary information
