# Supplementary material for: High-dimensional immunotyping of tumors grown in obese and non-obese mice
Source: Dis Model Mech. 2021 Mar 30;14(4):dmm048977. doi: 10.1242/dmm.048977 (PMC8033414; doi:10.1242/dmm.048977)
Supplement: Supplementary information [file dmm-14-048977-s1.pdf]

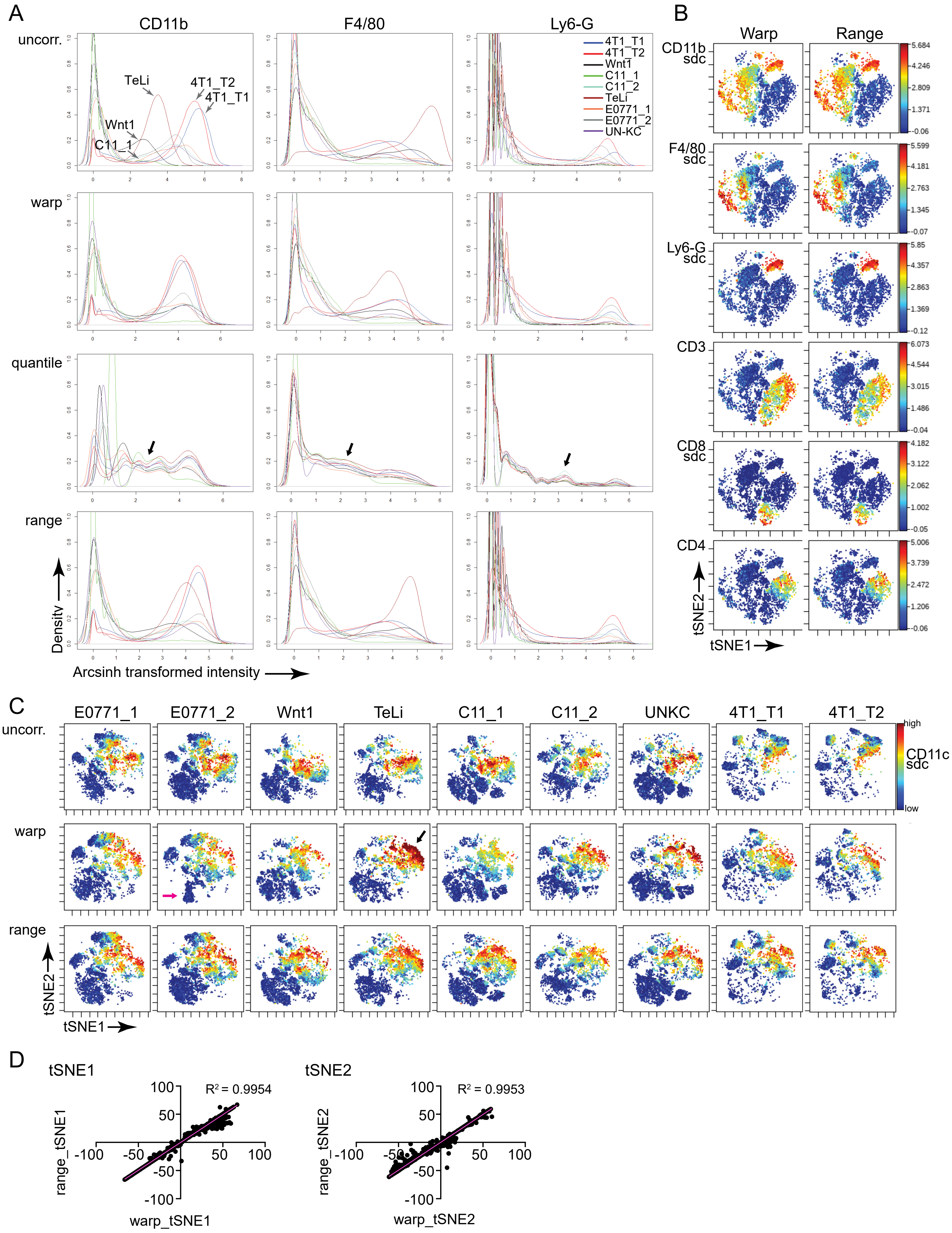

Disease Models & Mechanisms • Supplementary information

Fig. S1. Batch correction testing with 9 testing batches using two different panels with 18 common channels including shared different channels (sdc).

The 9 batches contain a total of 54 files (no HFD files) of CD45+ live cells with 5420 cells per file.

A. Cydar intensity distribution plots showing the third sample from all 9 batches. Intensities were plotted after arcsinh transformation for uncorrected, warp, quantile, and range corrected files. Three representative phenotyping channels are shown. Black arrows indicate the quantile density distribution that does not match the original uncorrected data.

B. Representative viSNE marker heat plots from 1 sample (E0771_1-C1) from a viSNE run where the warp and range corrected files for all 9 batches were run together. Range and warp plots are shown on the same scale; intensity heat scales vary between markers.

C. Three separate viSNE runs using the same seed (1794942912) were performed for each: uncorrected, warp corrected, and range corrected datasets. Black and pink arrows indicate warping artifacts. A representative sample was plotted for each batch.

D. Linear regression with R squared values comparing warp and range tSNE coordinates from data in panel B.

Disease Models & Mechanisms • Supplementary information

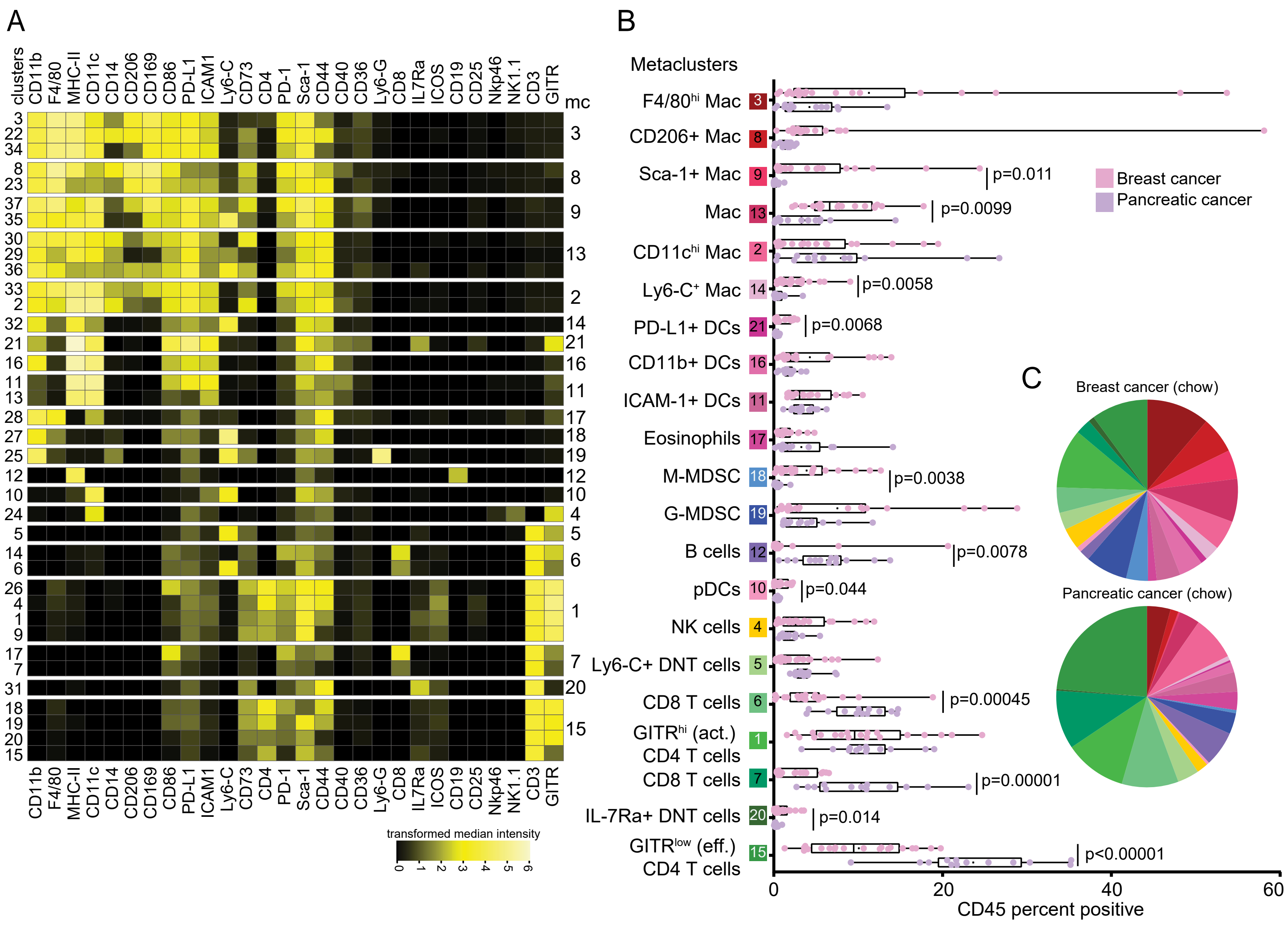

Fig. S2. Immune infiltrate metacluster abundance between breast and pancreatic cancer.

A. Heat map of arcsinh transformed batch corrected median marker intensity for the 37 clusters (left labels) and 28 markers (bottom labels). The 21 metaclusters are indicated by row spacing and numbers to the right. Row order is the same as in Figure 4A). N=57: E0771 (N=9/8), Wnt1 (N=6/6), TeLi (N=5/5), C11 (N=8/3), UN-KC (N=5/2).

B and C. Breast cancer N=20, Pancreatic cancer N=13.

B. Min to max box and whiskers plots with all points showing, median is the vertical line, dot is the mean. The plots show metacluster abundance from the combined breast cancer and pancreatic cancer non-obese murine tumors. Significant p values are displayed on the plot. T-tests were not adjusted, unpaired tests, with SD assumed.

C. Pie charts showing the relative mean abundance of the immune metaclusters between pancreatic and breast cancer tumor models (data from chow-fed non-obese murine tumors only).

Disease Models & Mechanisms • Supplementary information

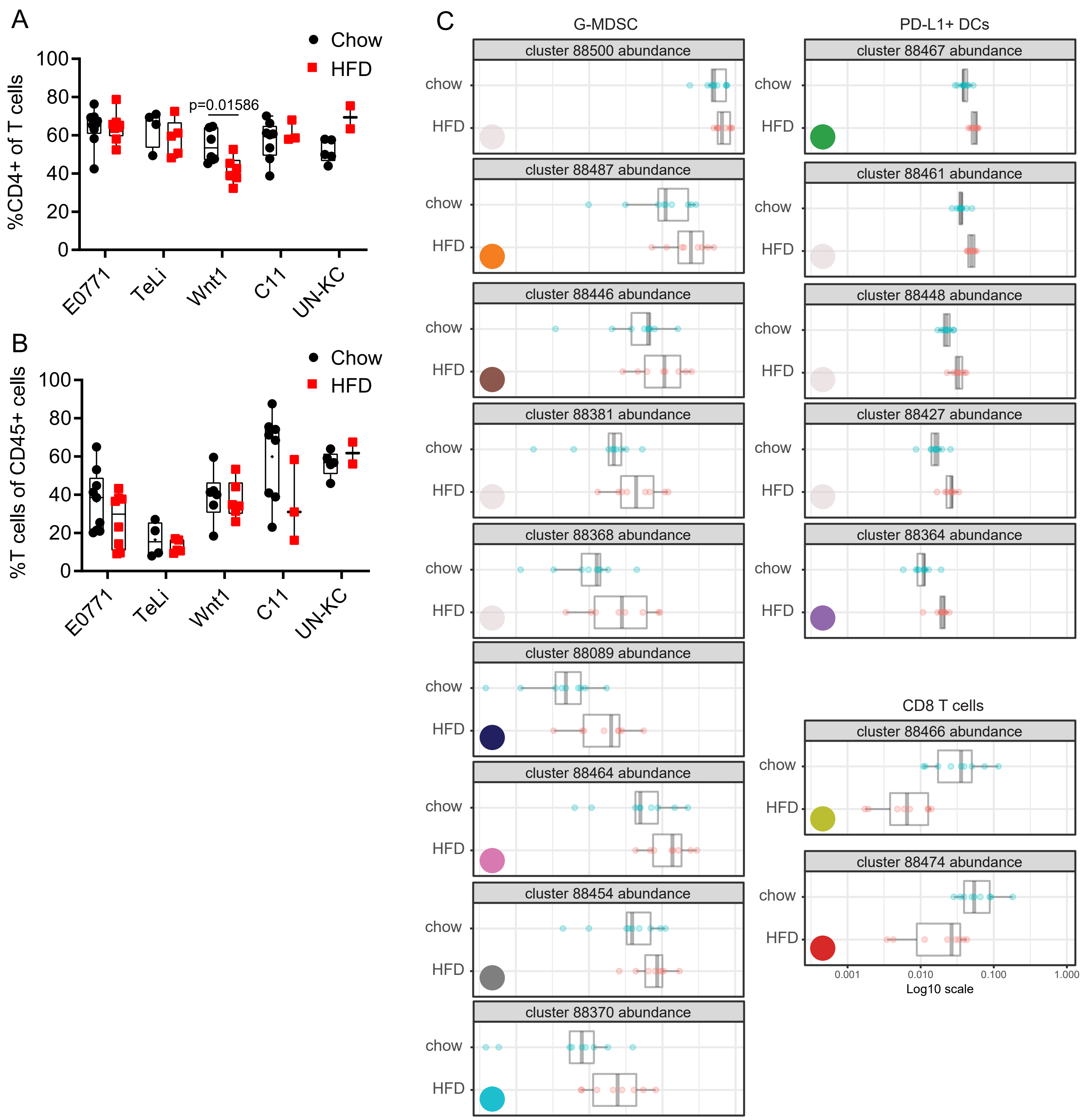

Fig. S3. T cell analysis and CITRUS significant cluster abundance.

A and B. Box and whiskers plots with all data points showing, (mean, min to max). T-tests were not adjusted, unpaired tests, with s.d. assumed between chow and HFD for individual cell subsets. E0771 (N=9/8), Wnt1 (N=6/6), TeLi (N=5/5), C11 (N=8/3), UN-KC (N=5/2).

A. Percent of CD4 T cells out of total T cells for all tumor models.

B. Percent of T cells out of total CD45+ immune infiltrating cells for all tumor models.

C Abundance plots for significant CITRUS clusters from E0771 SAM CITRUS analysis. Color-coding circle corresponds to Figure 6C, D. Plots were generated by Cytobank. E0771 (N=9/8).

Disease Models & Mechanisms • Supplementary information
